# Design and protocol for a cluster randomised trial of enhanced diagnostics for tuberculosis screening among people living with HIV in hospital in Malawi (CASTLE study)

**DOI:** 10.1371/journal.pone.0261877

**Published:** 2022-01-10

**Authors:** Rachael M. Burke, Saulos Nyirenda, Hussein H. Twabi, Marriott Nliwasa, Elizabeth Joekes, Naomi Walker, Rose Nyirenda, Ankur Gupta-Wright, Katherine Fielding, Peter MacPherson, Elizabeth L. Corbett

**Affiliations:** 1 Faculty of Infectious and Tropical Disease, Clinical Research Department, London School of Hygiene & Tropical Medicine, London, United Kingdom; 2 Malawi Liverpool Wellcome Clinical Research Programme, Blantyre, Malawi; 3 Zomba Central Hospital, Malawi Ministry of Health, Zomba, Malawi; 4 Helse Nord TB Initiative, Kamuzu University of Health Sciences, Blantyre, Malawi; 5 Department of Clinical Sciences, Liverpool School of Tropical Medicine, Liverpool, United Kingdom; 6 Department of HIV AIDS, Malawi Ministry of Health, Lilongwe, Malawi; 7 Institute for Global Health, University College London, London, England; 8 Faculty of Epidemiology and Population Health, Infectious Disease Epidemiology Department, London School of Hygiene & Tropical Medicine, London, United Kingdom; UNITED KINGDOM

## Abstract

**Background:**

People living with HIV (PLHIV) have a high risk of death if hospitalised in low-income countries. Tuberculosis has long been the leading cause of admission and death, in part due to suboptimal diagnostics. Two promising new diagnostic tools are digital chest Xray with computer-aided diagnosis (DCXR-CAD) and urine testing with Fujifilm SILVAMP LAM (FujiLAM). Neither test has been rigorously evaluated among inpatients. Test characteristics may be complementary, with FujiLAM especially sensitive for disseminated tuberculosis and DCXR-CAD especially sensitive for pulmonary tuberculosis, making combined interventions of interest.

**Design and methods:**

An exploratory unblinded, single site, two-arm cluster randomised controlled trial, with day of admission as the unit of randomisation. A third, smaller, integrated cohort arm (4:4:1 random allocation) contributes to understanding case-mix, but not trial outcomes. Participants are adults living with HIV not currently on TB treatment. The intervention (DCXR-CAD plus urine FujiLAM plus usual care) is compared to usual care alone. The primary outcome is proportion of participants started on tuberculosis treatment by day 56, with secondary outcomes of mortality (time to event) measured to to 56 days from enrolment, proportions with undiagnosed tuberculosis at death or hospital discharge and comparing proportions with enrolment-day tuberculosis treatment initiation.

**Discussion:**

Both DCXR-CAD and FujiLAM have potential clinical utility and may have complementary diagnostic performance. To our knowledge, this is the first randomised trial to evaluate these tests among hospitalised PLHIV.

## Introduction

Adults living with HIV who require admission to hospital in high-HIV prevalence, low-income countries have a very high risk of death with 31% pooled in-hospital mortality in a meta-analysis of African hospital cohorts 2007–2015 [1]. Advanced HIV and high mortality among people living with HIV (PLHIV) admitted to hospital is a persistent problem, despite improvements in antitretroviral thereapy (ART) regimens and widespread availability of ART at primary care [2]. Tuberculosis (TB) is the leading cause of hospital admission and inpatient death [3]. Suboptimal TB diagnostics contribute substantially to this problem: in an autopsy series meta-analysis, 48% of HIV positive people who died in hospitals in Africa had TB, and half of this was undiagnosed during life [4]. Two trials have demonstrated that these deaths are, at least in part, preventable: LAM-RCT [5] and STAMP [6] trials evaluated the first commercial urine lipoarabinomannan (LAM) lateral flow test for diagnosing TB among hospitalized people living with HIV (PLHIV). These trials showed increased TB diagnosis and reduced mortality in people with TB symptoms and other high-risk groups when urine TB diagnostics were used [5,6].

Urine testing for LAM using Determine TB LAM (AlereLAM, Alere/Abbott, USA) has been shown to reduce mortality when used in addition to sputum testing with Xpert MTB/Rif (Cepheid, USA) [5,6]. However, admission screening with sputum Xpert and AlereLAM will still fail to identify a substantial proportion of people who truly have TB. A study in Cape Town showed that, among 427 PLHIV sequentially admitted to inpatient wards, a third (139 people) had microbiologically confirmed TB when provided with enhanced mycobacterial-culture based diagnosis from multiple samples. Only 53% (73/139 people) had their TB diagnosed from urine LAM or sputum Xpert on samples collected in first 24 hours of admission [7].

Newer TB diagnostic tools hold promise to improve TB diagnosis and clinical outcomes among adults living with HIV admitted to hospital. The Computer Aided Screening for Tuberculosis in Low Resource Environments (CASTLE) trial will evaluate two new TB diagnostic tests used together. These tests are digital chest X-ray with Computer Aided Diagnosis (DCXR-CAD) [8,9] and a new high sensitivity urine LAM test (Fujifilm SILVAMP TB LAM [FujiLAM], Fujifilm Corporation, Japan) [10,11].

DCXR-CAD uses a computer software algorithm to analyze chest X-ray images and provide a probabilistic score of TB likelihood. Sensitivity and specificity compared to sputum Xpert or culture reference standard are similar that that of expert radiologists [8]. FujiLAM is a high sensitivity urine test for presence of LAM in urine, using a pair of monoclonal antibodies to LAM and novel silver amplification step. FujiLAM kits are self-contained lateral flow kits which require no electricity and no additional equipment. FujiLAM has been shown to be more sensitive than the AlereLAM (70.7% vs. 34.9% sensitive when compared to microbiological reference standard) [10,11].

Both of DCXR-CAD and FujiLAM appear promising in diagnostic accuracy studies, and using a combination of DCXR-CAD to sensitively screen for pulmonary TB disease and FujiLAM to detect disseminated TB is an attractive combination among inpatients. To our knowledge, there has only been one trial assessing patient relevant outcomes from use of DCXR-CAD (in outpatients in Malawi) [12] and no completed trials assessing outcomes from use of FujiLAM. Trials of clinical effectiveness of new diagnostic tools are important as the impact of clinicians’ testing practice, TB treatment decision-making and patient outcomes are unknown.

## Protocol

### Study design

The CASTLE Study is an unblinded, single site, two-arm (4:4 recruitment) cluster-randomised controlled trial, where clusters are day of admission. There is a third smaller enhanced diagnostic cohort that does not contribute to trial outcomes, so that the overall allocation ratio is 4:4:1 to usual care trial arm: intervention trial arm: diagnostic cohort, respectively. Table 1 summarises the trial schedule according to SPIRIT guidelines [13].

**Table 1:** CASTLE trial schedule (SPIRIT guidelines).

	STUDY PERIOD					
	Allocation	Enrollment	Post-allocation			End of participant f’up
TIMEPOINT**	8am each day	t = 0	t = 24 hours	*t = end of hospital admission (up to 56 days)*	*t = ~6–8 weeks*	*t = 56 days*
**ENROLMENT**:						
**Eligibility screen**		X				
**Informed consent**		X				
**INTERVENTIONS**:						
**INTERVENTION GROUP**: dCXR-CAD, urine LAM, sputum Xpert (if CAD high)		X				
**BOTH GROUPS**: Usual care (see description in manuscript)		X				
**BOTH GROUPS**: Sputum sample for culture		X				
**ASSESSMENTS**:						
On ART? Presence/absence TB symptoms? Was TB in differential diagnosis? Able to walk unaided?		X				
Started on enrollment day TB treatment?			X			
Started on TB treatment during hospital admission?				X		
Discharge from hospital alive vs. in-hospital death?				X		
Enrollment mycobacterial culture results (culture and identification takes 6–8 weeks)					X	
Alive or dead at 56 days?						X

### Study hypothesis

The CASTLE Study will test the hypothesis that TB screening using FujiLAM and DXCR-CAD for all adults living with HIV requiring admission for any reason to medical wards at hospital will increase the proportion of people started on TB treatment. As secondary objectives we will assess: time to death, measured up to 56 days from randomisation; the proportion of people with undiagnosed microbiologically confirmed TB at death or discharge from hospital (see below for study specific definition); and proportion starting TB treatment in 24 hours from enrolment—recognising that limited study power means that these two secondary outcomes are more properly considered Phase 2 than Phase 3.

### Study site and population

The CASTLE Study will be conducted at Zomba Central Hospital (ZCH), a combined secondary / tertiary hospital in Southern Region, Malawi, which serves both urban and rural populations and has sixty adult medical inpatient beds. This was the Malawi site of the STAMP trial of urine TB screening (using AlereLAM, conducted between 2015–2017) [6]. In STAMP Malawi arm the overall prevalence of microbiologically confirmed TB was 17% (113/656 people), in the group allocated to receive urine-based diagnostics.

Usual care TB diagnostics available at ZCH include sputum smear microscopy, Xpert MTB/Rif, urine AlereLAM and plain film radiography with radiographers and non-radiologist clinicians to interpret films: all these are available on clinician request. Comprehensive HIV care for inpatients is available and includes routine provider-initiated HIV testing and counselling for all admissions, and CD4 count and viral load on clinician request.

CASTLE study participants are HIV-positive (confirmed by review of patient held medical records or lateral flow testing) adults aged 18 years or older who are admitted to medical wards at ZCH for any reason. Exclusion criteria include people who: are currently taking TB treatment; have taken TB treatment in the past six months; are unable or unwilling to provide consent to be in the trial; and/or have been in admitted to wards for more than 18 hours at the time of recruitment to the trial.

### Randomisation, blinding, definition of clusters

Days will be randomly assigned in a 4:4:1 ratio to one of three arms. The arms are usual care, TB diagnostics intervention (DCXR-CAD and FujiLAM) and enhanced diagnostic arm.

Randomisation is done by computer algorithm, using varying block sizes (blocks are nine, 18 or 27 days), with allocations placed into sequentially numbered opaque, sealed envelopes. Participants will be recruited up to 3pm each day. People admitted to wards after 3pm will be eligible for recruitment on the following day, providing less than 18 hours has elapsed since their admission time. This is in order to be able to complete all study interventions on the same day as recruitment.

Because of the nature of the study and the interventions offered, it is not possible to mask participants or research assistants to allocation groups. However, as far as possible, the investigators will be blinded to allocation groups until final analysis and data will be cleaned without reference to trial arm allocation.

### Interventions

All participants (regardless of trial arm) will have a single sputum sample for mycobacterial culture using Mycobacterial Growth Indicator Tubes (Bactec MGIT, BD). Positive results will be communicated to participants and Zomba district TB team as soon as available.

#### Usual care arm

Participants allocated to usual care will receive clinician directed care, using any of the facilities available at ZCH (as above).

#### Intervention arm (DCXR-CAD and FujiLAM)

Participants allocated to the intervention arm will receive a DCXR with CAD score (CAD4TBv6.0, Delft, Netherlands) and urine FujiLAM (Fujifilm Corporation, Japan) and AlereLAM testing. If the CAD score is ≥ 60, a single sputum for Xpert testing will be collected by study team. The CAD score, urine LAM results and Xpert result (if CAD score high) will be recorded in the participant’s medical file and the chest X-ray image will be made available on a computer on the ward. This is in addition to usual care (as above).

#### Enhanced diagnostic cohort

The enhanced diagnostic cohort contributes to better understanding of case-mix, but not trial outcomes. Participants in the enhanced diagnostic cohort (one out of every nine cluster days) will receive DCXR-CAD, FujiLAM and AlereLAM. They will also have a blood sample drawn for CD4 cell count, HIV viral load (both of which are available, but neither of which are routine in usual care), bacterial blood culture and serum CrAg performed immediately with results reported back to clinical team as soon as available. Blood will be stored for batch testing for Pro-calcitonin (PCT), CRP, and HIV drug resistance mutations (if HIV virus detected). Since May 2021, all participants (in any arm) can opt into having a blood sample taken for targeted HIV viral load. Prior to May 2021 only participants in diagnostic cohort had blood samples taken by the trial team for HIV viral load, although HIV viral load measurement has always been available upon clinical request as part of usual care.

### Definitions

#### TB treatment initiation

TB treatment initation is as recorded in Malawi National TB register paper ledger at Zomba hospital. It is measured up to midnight on day of hospital discharge.

#### Time of TB treatment

Time TB treatment is dispensed from pharmacy to participant (or to the participant’s guardian or ward nurse).

#### Microbiologically confirmed TB

At least two positive Acid Fast Bacilli (AFB) smears or one or more Xpert Mtb/Rif positive or one or more culture positive for Mycobacterium tuberculosis on any specimen or a positive urine LAM result.

#### Undiagnosed TB

Refers specifically to participants who do not have a microbiological diagnosis of TB made on the basis of study or usual care samples, and have not been empirically started on TB treatment following a clinical diagnosis of TB, and have culture-positive Mycobacterium tuberculosis on study sputum culture taken at recruitment.

### Trial outcomes, assessment and analysis

The primary outcome is the proportion of participants started on TB treatment between recruitment into the trial and discharge from hospital, including on the same day as discharge. Analysis of the primary outcome will be done on an intention to treat basis, with all participants allocated to trial groups included and analysed in the group to which they were randomized.

The secondary trial outcomes will be time to death due to any cause, measured up to 56 days from enrolment in the trial; proportion of people starting TB treatment within 24 hours of enrolment in the trial; and proportion of people with undiagnosed microbiologically-confirmed TB at discharge from hospital (undiagnosed TB has a specific definition for this trial, above). The mortality outcome (the only outcome measured after discharge from hospital) will be ascertained predominantly by phonecalls to participants or a representative nominated by the participant, with home tracing for those who don’t respond to phonecalls or who don’t have a phone number.

Adjustment to effect estimates will be made to take account of clustering of outcome by day of admission. The full statistical analysis plan will be finalised before completion of enrolment, approved by the Data Safety and Monitoring Committee, and made publically available.

### Rationale for primary outcome

With TB being the most common cause of inpatient death among PLHIV, mortality provides the ideal outcome for TB diagnostic trials, as for the LAM-RCT and STAMP trials. For the single site CASTLE trial it is not feasible to recruit enough patients to be confident to have statistical power to detect a mortality benefit as primary outcome; instead the trial is focused on the more immediate and common outcome of increasing the proportion of inpatients started on TB treatment. TB treatment initiation is directly impacted by enhanced TB diagnostics, allowing a smaller trial comparing usual care and enhanced TB diagnostic arms. We consider this outcome as important for two main reasons. Firstly, whilst practices around empiric TB treatment (i.e. treatment in the absence of microbiological confirmation) vary substantially in different settings, in most settings TB is probably underdiagnosed among severely ill PLHIV in hospital. This is most notably demonstrated in autopsy series meta-analysis where 46% of people with TB were not diagnosed ante mortem, and similar findings in two more recent autopsy studies [4,14,15]. Secondly, both LAM-RCT and STAMP increased TB treatment initiation in the enhanced TB diagnostics arms, from 47% to 52% in LAM-RCT and from 15% to 22% in the urinary diagnostic arm of STAMP, consistent with a causal role in the reduced mortality [5,6]. As such, increasing TB treatment initiation is likely to be an important step towards further reduced mortality, provided that accuracy is maintained. TB treatment initiation is the primary outcome of the CASTLE trial, with mortality as a secondary outcome.

### Sample size

We plan to recruit 102 clusters per trial arm (i.e. 102 clusters in each of usual care and intervention arms), with approximately 306 participants per arm. We assume 10% of people in usual care arm will start TB treatment and hypothesise that the screening intervention could increase this to 18%. We assume a mean cluster size of three (i.e. three eligible participants admitted to hospital each day) and a coefficent of variation (k) of 0.005 (i.e. that clusters are relatively similar to each other). This will yield 82% power with 5% type 1 error to detect a difference between groups at least this large.

For the secondary mortality outcome, we hypothesise that 20% of participants will die within 56 days from enrolment. If the intervention time to death compared to usual care had hazard ratio of 0.6 and if there were a mean of four participants per cluster (i.e. four HIV-positive participants admitted to hospital per day) there would be 82% power to detect a mortality difference at least this large. If overall mortality is lower than 20%, the cluster size is smaller than four, or the effect size is lower power to detect a difference between arms is substantially lower.

In addition, we will recruit a further 26 clusters (approximately 78 people) in the enhanced diagnostic cohort, which will not contribute to trial outcomes.

### The enhanced diagnostic cohort

The first objective of the enhanced diagnostic cohort is to provide contextual information on the trial population, both laboratory tests such as CD4 counts and HIV viral load and more detailed clinical information about causes of admission to hospital and clinical course in hospital. The second objective is to investigate the prevalence of HIV viral failure, and infectious diseases leading to admission and contributing to deaths among PLHIV admitted to hospital in Zomba.

To put the HIV virological failure question into context; Malawi has a well performing national HIV programme and in 2020 it is estimated that 78% of all people living with HIV were established on antitretoroviral therapy (ART); this was 86% of all those who knew their HIV status [16]. In 2015–2017 in an analysis from the STAMP trial, 32% of PLHIV admitted to adult medical wards in ZCH had HIV virologic failure and the vast majority had resistance mutations to one or more first line ART drugs (at the time, first line ART was EFV+3TC+TDF). In 2019 the majority of PLHIV in Malawi were switched from non-nucleoside-reverse-transcriptase inihibitor (NNRTI) containing first line ART to integrase inhibitor containing ART (DTG+3TC+TDF), including those already stable on NNRTI containing regimens. As an exploratory aim, we intend to describe the prevalence of HIV virological failure among PLHIV admitted to hospital and the prevalence and types of drug resistance mutations in those with HIV virological failure, in the context of the roll out of dolutegravir containing ART.

### Funding, ethics and regulatory information

The sponsor of the trial is the London School of Hygiene & Tropical Medicine (rgio@lshtm.ac.uk). The protocol was given ethical approval by the ethics committess at the University of Malawi College of Medicine (COMREC, reference P.08/19/2772) and the London School of Hygiene & Tropical Medicine (LSHTM REC, reference 17799). All participants in the trial will be asked to give individual written informed consent. For people who are illiterate, they will give witnessed thumbprint consent. All participants are adults and there will be no proxy consent (i.e. lack of capacity to consent to the trial is an exclusion criterion).

The trial is registered at clinicaltrials.gov (NCT04545164). The trial is funded by the Wellcome Trust, through a PhD fellowship awarded to RMB (203905/Z/16/Z). The funder will have no role in the design or conduct of the trial. Any important trial amendments will be approved by the above ethics committees, communicated to sponsor and trial regisration will be updated. There is an independent Data Safety and Monitoring Committee (DMSC) comprised of three independent members, who have an advisory role for the trial. The Clinical Research Support Unit at Malawi Liverpool Wellcome Trust will periodically visit to review the study conduct. Both LSHTM REC and COMREC will receive annual monitoring reports.

No data are associated with this article. During the trial, data will be collected electronically on tablets using OpenDataKit. During the conduct of the trial and afterwards participant identifiable information will be kept confidential and held on a secure database in Malawi at the Malawi-Liverpool-Wellcome Trust. After completion of the trial, and at time of publication of the trial, anonymous trial data will be made available on LSHTM data compass.

The full protocol (version 5.1, date 2020-10-30), consent forms (S1 Appendix), DSMC charter (S2 Appendix) and SPIRIT checklist (S3 Appendix) [13] are available as an Supplementary Information to this article.

## Discussion

The CASTLE trial is a pragmatic cluster randomised trial of the effectiveness of the combination of two new TB diagnostic tests. Both tests have shown promise in diagnostic accuracy studies, are relatively affordable, and have quality certification (CE mark) from the European Union as diagnostic tests. However, to date, there are no studies of effect on patient-relevant outcomes in inpatients. People living with HIV admitted to hospital, particularly in low-income, high-HIV prevalence countries such as Malawi, have an unacceptably high risk of death. TB is the major cause of mortality and, as shown by STAMP and LAM-RCT trials [5,6], is at least partly preventable.

The CASTLE trial has some limitations. The main limitation is the lack of a robust microbiological reference standard for ascertaining which participants truly have TB, despite taking a single research culture sample. This is particularly relevant for the outcome of “undiagnosed TB at discharge”. TB is extremely difficult to fully refute, as evidenced by both autopsy findings (half of all TB diagnosed among PLHIV in autopsy series meta-analysis was not diagnosed antemortem despite the people who died having been in contact with health services prior to death) [4,14,15], and that in clinical practice TB is often treated “empirically” (i.e. without microbiological confirmation) [17]. Anticipating that this intervention may increase the amount of empiric TB treatment used, we chose to have one study sputum culture as a minimum objective TB reference, whilst acknowledging that a single sputum culture result is far from a definitive way to diagnose or refute TB. However, diagnostic accuracy studies with extensive microbiological reference testing have already been undertaken to evaluate both the diagnostic tests used, and the focus of this trial is the impact of introducing these tests on patient-relevant outcomes.

We cannot see any feasible way to have introduced masking in this trial, as clinicians will be able to review laboratory results and chest X-ray images. The outcomes for the trial (as with almost any diagnostic intervention) depend—to an extent—on healthcare provider behaviour subsequent to test results. It is possible that there may be an effect of clinician behaviour for participants in usual care arm, particularly through requesting more usual care TB tests. This may reduce the difference between arms compared to if fewer TB tests (e.g. AlereLAM, conventional chest Xray and sputum Xpert) were requested in usual care clusters. However, the effect of interest for the trial is the additional impact of DCXR-CAD plus FujiLAM over and above maximising the use of existing diagnostic tests.

The cluster randomised design (by admission day) is designed to enable batching of study tasks (for example, radiographer time) and to allow the trial to recruit a larger number of participants than might otherwise be possible with existing resources. It is also possible that clustering patients by day might be less likely to alter clinician behaviour for participants in the usual care only arm than individual randomisation, as clinicians will not be dealing with patients having had different TB testing simultaneously on the same admission ward round.

In summary, people living with HIV admitted to hospital in low income high-HIV-prevalence settings have a high risk of death and TB is the leading cause of both admission and death. The CASTLE trial is a pragmatic single site cluster randomised trial of the effectiveness of enhanced TB diagnostics offered to all PLHIV admitted to hospital using DCXR-CAD plus FujiLAM. We are hopeful that the CASTLE trial will provide policy-relevant evidence of the impact and effectiveness of introducing new TB diagnostic tools in order to move beyond diagnostic accuracy studies alone.

## Supporting information

S1 Fig CASTLE trial schedule (SPIRIT guidelines). (DOCX)

S1 Appendix CASTLE study protocol. (PDF)

S2 Appendix CASTLE data safety and monitoring committee charter. (PDF)

S3 Appendix CASTLE trial protocol SPIRIT checklist. (DOCX)
