# Supplementary material for: Design and protocol for a cluster randomised trial of enhanced diagnostics for tuberculosis screening among people living with HIV in hospital in Malawi (CASTLE study)
Source: PLoS One. 2022 Jan 10;17(1):e0261877. doi: 10.1371/journal.pone.0261877 (PMC8746787; doi:10.1371/journal.pone.0261877)
Supplement: S1 Fig — (DOCX) [file pone.0261877.s001.docx]

Figure 1: CASTLE trial schedule (SPIRIT guidelines)

|  | **STUDY PERIOD** | | | | | |
| --- | --- | --- | --- | --- | --- | --- |
|  | **Allocation** | **Enrollment** | **Post-allocation** | | | **End of participant f’up** |
| **TIMEPOINT**** | 8am each day | t=0 | t=24 hours | *t=end of hospital admission (up to 56 days)* | *t= ~6-8 weeks* | *t=56 days* |
| **ENROLMENT:** |  |  |  |  |  |  |
| **Eligibility screen** |  | X |  |  |  |  |
| **Informed consent** |  | X |  |  |  |  |
| **INTERVENTIONS:** |  |  |  |  |  |  |
| **INTERVENTION GROUP:** dCXR-CAD, urine LAM, sputum Xpert (if CAD high) |  | X |  |  |  |  |
| **BOTH GROUPS:** Usual care (see description in manuscript) |  | X |  |  |  |  |
| **BOTH GROUPS:** Sputum sample for culture |  |  |  |  |  |  |
| **ASSESSMENTS:** |  |  |  |  |  |  |
| On ART? Presence/absence TB symptoms? Was TB in differential diagnosis? Able to walk unaided? |  | X |  |  |  |  |
| Started on enrollment day TB treatment? |  |  | X |  |  |  |
| Started on TB treatment during hospital admission? |  |  |  | X |  |  |
| Discharge from hospital alive vs. in-hospital death? |  |  |  | X |  |  |
| Enrollment mycobacterial culture results (culture and identification takes 6-8 weeks) |  |  |  |  | X |  |
| Alive or dead at 56 days? |  |  |  |  |  | X |
