# Supplementary material for: Design and protocol for a cluster randomised trial of enhanced diagnostics for tuberculosis screening among people living with HIV in hospital in Malawi (CASTLE study)
Source: PLoS One. 2022 Jan 10;17(1):e0261877. doi: 10.1371/journal.pone.0261877 (PMC8746787; doi:10.1371/journal.pone.0261877)
Supplement: S1 Appendix — (PDF) [file pone.0261877.s002.pdf]

The CASTLE study

Computer Aided Screening for Tuberculosis in Low Resource Environments

Document: Study protocol

Version: 5.1

Author: Dr Rachael Burke

Date modified: 2020-10-30

Last modified by: Dr Rachael Burke

TABLE OF CONTENTS

1. REGULATORY INFORMATION **3**
2. ABSTRACT / EXECUTIVE SUMMARY **5**
3. ABBREVIATIONS **7**
4. BACKGROUND AND RATIONALE **8**
4.1. Inpatients living with HIV 8
4.2 Study rationale 12
5. RESEARCH QUESTIONS, AIMS AND OBJECTIVES **13**
5.1 Research Questions 13
5.2 Objectives 13
6. STUDY DESIGN **16**
7. STUDY POPULATION **17**
7.1. Trial Participants 17
7.2. Inclusion Criteria 17
7.3. Exclusion Criteria 17
8. RECRUITMENT, RANDOMISATION AND BLINDING **18**
8.1. Randomisation, allocation and masking of clusters (days) 18
8.2 Screening and recruitment of participants 18
8.3 Informed Consent 19
9. INTERVENTIONS, METHODS AND PROCEDURES **20**
9.1 Baseline Assessments and Procedures (trial arms) 20
9.2 Baseline assessments and procedures (enhanced diagnostic observational arm) 20
9.3 TB Definitions 21
9.4 Definitions of clinical / microbiological diagnoses (enhanced diagnostic arm only) 22
9.5 Training for responsible medical team 22
9.6 Subsequent procedures (trial arms) 22
9.7 Subsequent procedures (enhanced diagnostic observational arm only) 23
9.8 Storage of specimens 24
9.9 Pilot phase procedures 24
9.10 Study timeline 25
10. OUTCOME EVALUATION **26**
10.1 Follow-up procedures and ascertainment of outcomes (trial arms) 26
10.2 Follow-up procedures and ascertainment of outcomes (enhanced diagnostic observational arm) 26
10.3 Discontinuation/Withdrawal of Participants 26
10.4 Definition of End of Trial 26
11. SAFETY REPORTING **27**
12. STATISTICS AND SAMPLE SIZE JUSTIFICATION **28**
12.1 Assumptions 28
12.2 Sample size and power (primary outcome) 28
12.3 Sample size and power (secondary outcomes) 28
12.4 Data analysis plan 29
13. DATA MANAGEMENT **31**
13.1 Data Recording and Record Keeping 31
14. MONITORING AND QUALITY ASSURANCE **32**
14.1 Data Safety Monitoring Committee 32
14.2 Trial Investigator Team 32
14.3. Ethical Considerations and Approvals 33
14.4 Possible constraints 35
15. BUDGET AND INSURANCE **36**
15. PUBLICATION POLICY **39**
16. REFERENCES **40**
APPENDIX 1: PREVIOUS STUDIES OF DCXR-CAD **42**
APPENDIX 2: CLINICAL AND CLINICAL / MICROBIOLOGICAL DEFINITIONS **45**

Approved by
College of Medicine
10-Dec-2020
(COMREC)
Research and Ethics Committee

1. REGULATORY INFORMATION

Title: Computer Aided Screening for Tuberculosis in Low Resource Environments

Short title: CASTLE study

Research Organisation Ref: LOI 18.159 (LSHTM), LOI 510 (MLW)

Ethics Reference: 17799 (LSHTM), 2772 (COMREC)

Trial Registration Number: pending

Date and Version No: Version 5.1 2020-10-30

Principal Investigator: Dr Rachael Burke, Department Clinical Research, Faculty of Infectious and Tropical Disease, London School of Hygiene & Tropical Medicine

Sponsor: The London School of Hygiene & Tropical Medicine is the main research sponsor for this study. For further information regarding the sponsorship conditions, please contact the Research Governance and Integrity Office:
London School of Hygiene and Tropical Medince
Keppel Street
London WC1E 7HT
+44 2079272626
rgio@lshtm.ac.uk

Funder: Wellcome Trust.

Conflict of interests: We declare no conflicts of interest

Compliance: The trial will be conducted in compliance with the protocol, ICH GCP Guidelines and other relevant regulatory requirements applying in the countries in which the trial will be conducted.

Approved by
College of Medicine
10-Dec-2020
(COMREC)
Research and Ethics Committee

Key trial investigators and contacts:

Role	Name	Institution	Contact Details
Principal Investigator	Dr Rachael Burke	London School of Hygiene & Tropical Medicine	Rachael.burke@lsthm.ac.uk<br>+44 77295058417
PhD supervisor / co-investigator	Prof Liz Corbett	London School of Hygiene & Tropical Medicine	Liz.corbett@lshtm.ac.uk<br>+ 265 999981439
PhD supervisor / co-investigator	Dr Peter MacPherson	Liverpool School of Tropical Medicine	peter.macpherson@lstmed.ac.uk<br>+265 99 717 6230
PhD supervisor / co-investigator	Dr Ankur Gupta-Wright	London School of Hygiene & Tropical Medicine	Ankur.gupta-wright@lshtm.ac.uk
Co-investigator	Prof Katherine Fielding	London School of Hygiene & Tropical Medicine	Katherine.Fielding@lshtm.ac.uk
Co-investigator	Dr Naomi Walker	London School of Hygiene & Tropical Medicine	Naomi.walker@lshtm.ac.uk
Co-investigator	Dr Marriot Nilwasa	College of Medicine, Malawi	mnliwasa@gmail.com
Co-investigator	Dr Saulos Nyirenda	Head, Department of Medicine, Zomba Central Hospital	Saulos.nyirenda@yahoo.com
Co-investigator	Dr Elizabeth Joekes	Liverpool School of Tropical Medicine	e.joekes@liverpool.ac.uk
Co-investigator	Rose Nyirenda	Director, Department HIV / AIDS	nyirendarose@gmail.com
Sponsor	LSHTM Research Governance Office.	London School of Hygiene & Tropical Medicine.<br>Keppel Street, London. WC1E 7HT.	rgio@lshtm.ac.uk

Collaborator	Dr James Mpunga	Director, National TB Programme	
Collaborator	James Kandulu	Director Diagnostics, Ministry of Health	

Approved by
College of Medicine
10-Dec-2020
(COMREC)
Research and Ethics Committee

2. ABSTRACT / EXECUTIVE SUMMARY

Trial title	Computer Aided Screening for Tuberculosis in Low Resource Environments (CASTLE)
Short title	CASTLE study
Trial Design (methodology)	Single site (Zomba Central Hospital) cluster randomised trial with two trial arms and a third nested observational enhanced diagnostic cohort that will not contribute to trial outcomes (4:4:1 allocation, randomised by admission day).
Trial population	HIV infected adult patients requiring admission to medical wards at Zomba Central Hospital. Unit of randomisation will be admission day.
Planned sample size	102 clusters per trial arm (approximately 306 participants). A further 26 clusters in enhanced diagnostic cohort (approximately 78 participants). Total of 230 clusters with approximately 690 participants.
Follow up duration	56 days (eight weeks) from day of recruitment
Recruitment period	January 2020 – March 2021.
Trial intervention	Digital Chest x-ray with Computer Aided Diagnosis (DCXR-CAD) and urine high sensitivity lipoarabinomannan (FujiLAM) screening performed on first day of admission on participants admitted on days assigned to trial intervention arm. Numerical X-ray TB score and interpretation ("Pulmonary TB likely" or "Pulmonary TB not likely"), and FujiLAM results, appended into patient's notes. X-ray imaging available for clinical team review on study computer in order to inform TB treatment decision making. If a participant's CAD score indicates "TB likely", they will have sputum taken for Xpert Mtb/Rif. DCXR-CAD is in addition to usual care in the intervention arm. The control arm is assigned to usual care alone.

	Objective	Outcome Measures / Endpoints
1. Primary	To determine the effect of DCXR-CAD plus FujiLAM plus usual care vs. usual care alone on;<br>1.1 TB treatment initiations	1.1 Proportion of participants starting TB treatment during course of inpatient stay (censored at 56 days)
2. Secondary	To determine the effect of DCXR-CAD plus FujiLAM plus usual care vs. usual care alone on;<br><br>2.1 Mortality (time to event)<br>2.2 Undiagnosed TB<br>2.3 Same day TB treatment initation	2.1 Time (in days) to death from any cause, with censoring at 56 days.<br><br>2.2 Proportion of participants who are culture positive for *M. tuberculosis* (M.tb) in sputum, who are not started on TB treatment at the time of discharge from hospital or are current inpatients not on TB treatment by the time of culture result being made available.<br>2.3 Proportion of participants starting TB treatment within 24 from from time of recruitment.
3.Pre-planned analyses.	To determine the effect of DCXR-CAD plus FujiLAM plus usual care vs. usual care alone on;<br>3.1 Inpatient mortality (proportion of participants dying as inpatients).	3.1 Proportion of participants dying during admission (censored at 56 days) from any cause.

Approved by College of Medicine 10-Dec-2020 (COMREC) Research and Ethics Committee

	3.2 Total mortality (proportion experiencing death)<br><br>3.2 Proportion of participants with a TB diagnosis that is microbiologically confirmed vs. clinically diagnosed.<br><br>3.3 To determine intervention fidelity in this setting.<br><br>3.4 To determine diagnostic accuracy of DCXR-CAD in an inpatient, HIV positive setting.	3.2 Proportion of participants dying up to 56 days from any cause.<br><br>3.2 Proportion of participants started on TB treatment with (a) microbiologically confirmed TB vs. (b) clinically or radiologically diagnosed TB.<br><br>3.3 Proportion of participants randomised to DCXR-CAD plus FujiLAM arm who received DCXR-CAD and had a urine FujiLAM result.<br><br>3.4 Sensitivity, specificity, PPV and NPV for CAD score compared to a composite microbiological reference standard and a clinical standard.
4. Other objectives	To describe the range of pathology among people living with HIV requiring admission to hospital (in diagnostic cohort)<br><br>To describe the prevalence of HIV virological failure in hospital, and HIV viral resistance in people with virological failure.	Descriptive statistics.<br>See section 5.2.

Dissemination of findings	Data will be disseminated to COMREC, local institutions, academic bodies and professional associations within Malawi and internationally (for example, STOP TB partnership). Data will be published in a timely manner in peer reviewed journals. Feedback on results will also be given to Zomba hospital staff and the Department of Medicine, the district TB officers in Zomba district, and the Malawi National HIV programme.

Trial schematic

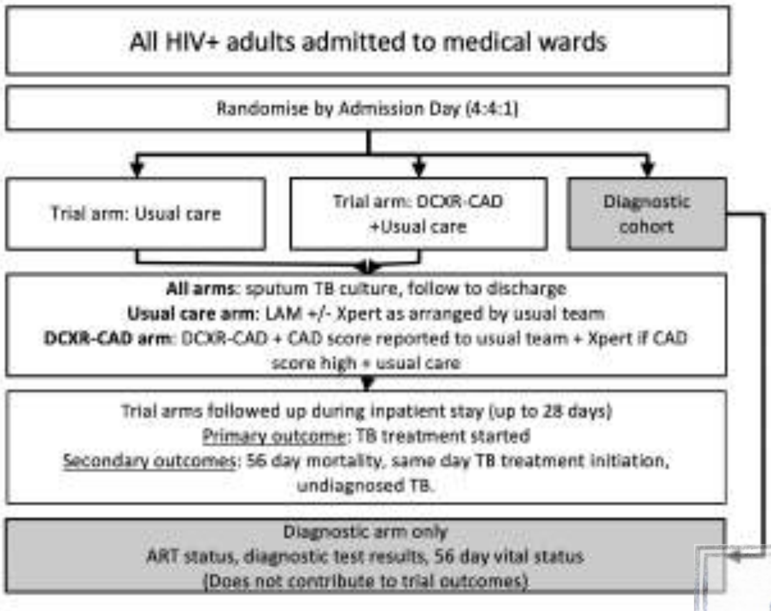

Approved by College of Medicine
10-Dec-2020
(COMREC)
Research and Ethics Committee

3. ABBREVIATIONS

ART	Anti-retroviral therapy or treatment.
CrAg	Cryptococcal Antigen
CRF	Case Report Form
CRP	C-reactive protein
CAD	Computer Aided Diagnosis
COM	College of Medicine
COMREC	College of Medicine Research Ethics Committee
(D)CXR	(Digital) Chest x-ray
DCXR-CAD	Digital Chest x-ray with Computer Aided Diagnosis
FujiLAM	Refers to a high sensitivity lipoarabinomannan TB screening assay manufactured by FujiFilm
HIV	Human Immunodeficiency Virus
ICF	Informed Consent Form
ICH GCP	International Committee Harmonisation Good Clinical Practice
IRIS	Immune Reconstitution Inflammatory Syndrome
LAM	Lipoarabinomannan (a urine based screening test for TB)
LSTHM	London School of Hygiene and Tropical Medicine
LSTM	Liverpool School of Tropical Medicine
NPV	Negative Predictor Value
MLW	Malawi-Liverpool-Wellcome
MTA	Material Transfer Agreement
M. tb	*Mycobacterium tuberculosis*
PJP (or PCP)	*Pneumocystsis jiorevci* pneumonia
PCT	Procalcitonin
PIL	Participant Information Leaflet
PITC	Provider Initiated Counselling and Testing
PLHIV	People living with HIV
PPV	Positive predictor value
(S)AE	(Serious) Adverse Event
STAMP	Refers to a clinical trial of urine LAM among PLHIV (Rapid urine-based screening for tuberculosis in HIV positive patients admitted to hospital in Africa: a pragmatic, multicentre, parallel-group, double-blind, randomised controlled trial, *Gupta Wright* et al, Lancet, 2018)
TB	Tuberculosis
TDM	Therapeutic drug monitoring
WHO	World Health Organisation

Approved by
College of Medicine
10-Dec-2020
(COMREC)
Research and Ethics Committee

4. BACKGROUND AND RATIONALE

4.1. Inpatients living with HIV

Inpatient mortality

People living with HIV who present to hospitals and require admission in WHO AFRO region have an extremely high inpatient mortality rate. A meta-analysis of published African inpatient PLHIV cohorts from 2007 to 2011 showed in hospital mortality of 31%.[1]

Local Malawi data from the STAMP trial cohort of PLHIV admitted to Zomba Central Hospital 2015 – 2017 show overall inpatient mortality of 11.3% and 56-day mortality of 21% in the intervention group with urine LAM screening (by Alere Determine LAM) [reference 2, including unpublished data]. [2]

High TB prevalence

TB prevalence is likely to be high in hospitalized patients living with HIV in Southern and Eastern Africa. Autopsy studies have shown pooled TB prevalence of 43% among nine studies from adults with HIV from WHO AFRO countries. [3] In a study in Cape Town, a third of PLHIV admitted to inpatient wards, regardless of symptoms, had microbiologically confirmed TB when provided with enhanced culture based diagnosis from multiple samples. [4] A meta-analysis of hospitalized PLHIV in WHO AFRO region, relying on routine diagnostics and clinical suspicion, reported that 17% of all admissions were due to TB, making this the leading cause of admission among PLHIV. [5]

People with HIV and TB who are admitted to hospital have a high mortality rate. In the STAMP trial cohort in Zomba, Malawi, mortality at 56 days in people with microbiologically confirmed TB was 31%, compared to 18% among people without diagnosed TB. [2] In a meta-analysis of 160,647 PLHIV admitted to hospital worldwide, 29% admitted those due to TB died prior to discharge from hospital.[5]

Current difficulties with TB diagnosis

TB – particularly when associated with HIV – is difficult to diagnose, with the main problem being low sensitivity of currently-available diagnostic tests.

Nucleic acid amplification tests on sputum using Xpert MTB/Rif are recommended by WHO as the diagnostic tool of choice among PLHIV. Xpert MTB/rif is highly specific (98%) and reasonably sensitive (79% sensitive in PLHIV) compared to sputum culture. [6] However, using Xpert has not been shown to reduce mortality compared to using smear microscopy. [7] Furthermore, inpatients (and especially HIV-positive and seriously ill inpatients) often find it difficult to produce sputum, meaning that diagnostics based on a more readily available specimen would be beneficial. [2,4]

Rapid testing using lateral flow urine tests for lipoarabinomannan (LAM) has been shown to be specific for TB and sensitive for disseminated TB in immunosuppressed people with HIV (LAM diagnostic accuracy). [8] The STAMP trial showed that urine Alere LAM screening led to a non-significant reduction in all-cause mortality at 56 days among inpatients with HIV from 21% to 18%, and a significant mortality reduction in three pre-defined subgroups (those where TB was in differential diagnosis at admission, low CD4 count and low haemoglobin). [2]

Approved by
College of Medicine
10-Dec-2020
(COMREC)
Research and Ethics Committee

However, in a study in Cape Town, urine Alere LAM and sputum Xpert on samples obtained in the first 24 hours of admission still failed to identify nearly 50% of patients who had microbiologically confirmed TB on the basis of extended microbiological sampling across the course of admission. [4]

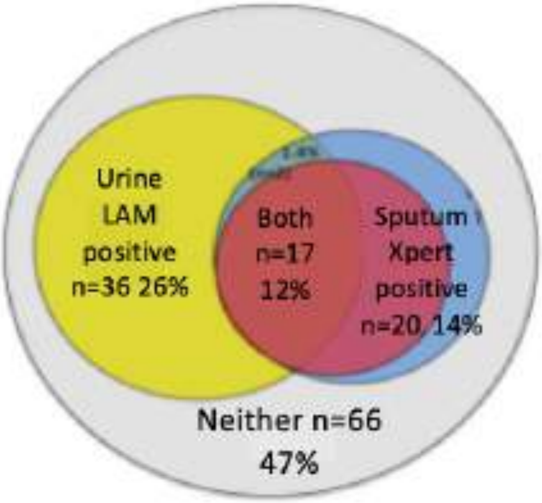

Figure 1: Venn diagram showing urine LAM (yellow circle) and sputum Xpert MTB/rif positives (blue circle) from samples obtained in 1st 24 hours of admission from 139 PLHIV inpatients with microbiologically confirmed TB (grey circle). Red circle represents sputum smear positive. Reference (4).

High sensitivity LAM

The STAMP trial (and other trials) used a "first generation" LAM assay manufactured by Alere / Abbott (USA). This is the LAM test that has been successfully rolled out across Malawi and other countries.

A new "second generation" high sensitivity LAM assay has recently been developed by FujiFilm corporation (Japan). This FujiLAM has superior sensitivity to the older Alere LAM. A study in South Africa of parallel testing of stored urine samples from hospitalised PLHIV showed that AlereLAM was 42% sensitive whereas FujiLAM was 70% sensitive when compared with a composite microbiological reference standard. [8]

This increased sensitivity means that FujiLAM has the potential to substantially improve the diagnosis of TB and improve patient outcomes even more that the already successful Alere LAM.

FujiLAM has a CE mark as an in vitro diagnostic test (IVD) by the EEA (European Economic Area.

Chest x-ray for TB diagnosis

Chest x-ray is recommended by WHO for TB diagnosis, including for clinical diagnosis of TB if microbiological tests are unavailable or negative. [9] Chest x-ray has high sensitivity for pulmonary TB, even in HIV co-infection, and continues to play an important role in TB diagnosis in high-income settings. Although chest x-ray has been used for many years as a diagnostic tool, widespread implementation in low-resource / high TB prevalence settings has been limited by poor access to high quality equipment and expert radiologists, low specificity (leading to over-diagnosis of TB if chest x-ray alone is used) and high inter-reader variability. [9]

Approved by College of Medicine
10-Dec-2020
(COMREC) Research and Ethics Committee

Chest x-ray can be used at several different stages of a TB diagnostic algorithm with diagnostic performance characteristics that depend on the setting. For PLHIV who are unwell and require hospital admission WHO recommends chest x-ray (if available) as part of the initial diagnostic work up. Chest x-ray is further recommended for people who are Xpert MTB/Rif negative or where Xpert MTB/Rif is unavailable as part of further investigations for TB, in conjunction with empiric TB treatment (see algorithm below). [10]

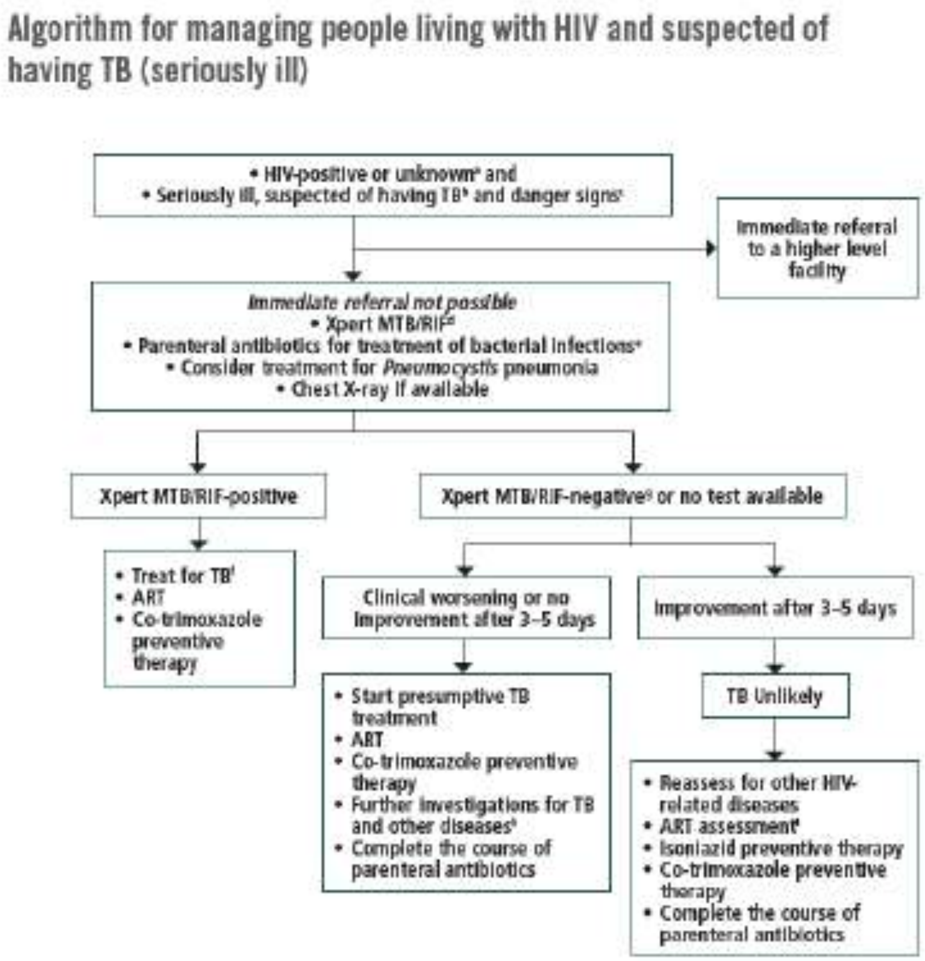

Figure 2. WHO algorithm for PLHIV who are seriously unwell (i.e. requiring hospital admission) and suspected of having TB from 2016 Consolidated guidelines on the use of antiretroviral drugs for treating and preventing HIV infection (reference (10)).

Currently, countries such as Malawi have low coverage of radiology services, including lack of trained radiologists. For example, in the STAMP trial in Zomba 2015 – 2017 only 23% of PLHIV admitted to hospital had a chest x-ray. [2]

CAD for Chest x-ray interpretation

Computer-assisted detection (CAD) software for chest x-ray interpretation – artificial intelligence algorithms used to classify digital images - is now available, and can be integrated within new digital x-ray units to provide immediate interpretation. [11 12 13 14 15 16 17 18 19]

A recent systematic review of available evidence for one CAD system (CAD4TB, Delft Imaging Systems, Netherlands) showed that sensitivity was as high as reading by radiologists, although

Approved by College of Medicine 10-Dec-2020 (COMREC) Research and Ethics Committee

specificity was slightly lower. [12] WHO recommends that *"CAD can be used for TB detection for research, ideally following a protocol that contributes to the required evidence base for guideline development".* [9]

Appendix 1 summarises existing literature on DCXR-CAD for TB diagnosis. There are no completed prospective studies on clinical impact of CAD, although one outpatient prospective study is ongoing in Malawi (PROSPECT study). [20] There are no ongoing or completed studies investigating effectiveness in inpatient populations.

Opportunistic infections, co-morbid conditions other than TB and HIV virological failure

Whilst TB is the leading cause of hospitalization and mortality in people living with HIV, it is clearly not the only cause of mortality.

In general, the epidemiology of prevalent conditions causing hospital admission among PLHIV in Southern and Eastern Africa has been sparsely described. In part this is due to the low availability of laboratory diagnostics in many countries in this region.

With the widespread availability of ART, an increasing proportion of people living with HIV admitted to hospital in Southern and Eastern Africa are immunosuppressed but ART experienced – either current ART users or having interrupted ART use. [21] [22] The WHO guidelines for public health management of advanced HIV states "the evidence supporting the package of interventions for people with advanced disease is derived from studies of ART-naïve patients" and that "further research is required to evaluate the optimal package of interventions to people presenting with treatment failure". [23]

In addition to TB, leading causes of mortality are likely to include sepsis syndromes, bacterial pneumonia, cryptococcal disease and pneumocystis jirovecci pneumonia (PJP), as well as non-infectious causes (for example, heart failure). [1] [24]

The 'ART era' of HIV care in Southern and Eastern Africa is characterized by a high coverage of ART at a population level, but persisting challenges related to treatment interruption or treatment failure. A robust understanding of the epidemiology of prevalent disease among PLHIV admitted to hospital in the 'ART era' is important in order to begin to design a package of interventions to reduce mortality. [23]

Data from STAMP study, also at Zomba Central Hospital in Malawi showed that 32% of PLHIV admitted to Zomba hospital 2015 – 2017 had HIV virological failure (HIV viral load >1000 copies/mL). 82% of people with HIV virological failure had resistance to two or more ART medicines. [25] Since 2017, the Malawi National HIV programme has switched from Efavirenz (from NNRTI class of medicines) to Dolutegravir (integrase inhibitor class) as first line ART. Dolutegravir is reported to have a higher barrier to resistance than NNRTIs. However, in a small study in Chiradzulu, 2 / 3 people with HIV virological failure on dolutegravir-containing ART had dolutegravir resistance mutations. [26]

In order to guide clinical practice and national guidelines it is important to have an overview of the prevalence of HIV virological failure and HIV viral resistance among PLHIV requiring hospital admission following the national switch to dolutegravir-containing ART.

Approved by College of Medicine 10-Dec-2020 (COMREC) Research and Ethics Committee

4.2 Study rationale

The need for studies of clinical effectiveness

Although chest x-ray is recommended by the WHO for TB screening and diagnosis in unwell patients in hospital, there is no clear evidence base supporting this recommendation. Empiric TB treatment is common in this setting, and is recommended by WHO in certain circumstances. [10]

Whilst DCXR-CAD for TB diagnosis has similar performance characteristics to a trained human reader, it is not known how systematic chest radiography combined with CAD interpretation will affect clinician's testing practice, rates of TB treatment, and patient outcomes. Therefore a study of clinical impact on patient-important outcomes of this intervention is required.

Were DCXR-CAD shown to be effective in this population, it has the potential to be used at scale in hospitals across Southern and Eastern Africa. Robust and relatively low-cost x-ray units are now available, and published estimated costs of DCXR-CAD are $1.46 per x-ray for TB screening on an outpatient clinic basis (inclusive of cost of x-ray unit over its lifetime, maintenance of x-ray unit, CAD license fee and radiographer time). [19]

Urine LAM screening using Alere LAM has been shown to improve patient outcomes in two trials. There is good evidence that FujiLAM is substantially more sensitive than Alere LAM. However for national TB/HIV programmes and guideline development it is important to know whether use of the more sensitive test leads to an improvement in clinical outcomes. Were FujiLAM shown to be more effective than Alere LAM in this population, it has the potential to replace Alere LAM and be rapidly rolled out by national programmes.

An important factor in the design of the present study is the strategy of investigating **<u>all</u>** patients regardless of whether TB is suspected or not: post-mortem studies show that a substantial burden of TB remains clinically unsuspected and is therefore not empirically treated. [3] We know that symptom screening for TB among PLHIV being admitted to hospital is neither sensitive nor specific. [2 27]

Approved by
College of Medicine
10-Dec-2020
(COMREC)
Research and Ethics Committee

5. RESEARCH QUESTIONS, AIMS AND OBJECTIVES

5.1 Research Questions

- Does implementation of systematic DCXR-CAD plus FujiLAM screening for TB, in combination with usual care diagnostics, in adults living with HIV being admitted to hospital increase the number of adults started on TB treatment compared to usual care alone?

- Is there a suggestion that implementation of systematic DCXR-CAD plus FujiLAM screening for TB, in combination with usual care diagnostics, in adults living with HIV being admitted to hospital reduces mortality by eight weeks, reduces undiagnosed TB or increases the number of people started on TB treatment on same day of admission?

- What are the clinical and microbiological characteristics of adults living with HIV admitted to hospital in a low resource area with a high coverage of dolutegravir based ART?

5.2 Objectives

Objectives	Outcome measures / endpoints
**Primary objectives**	
1.1 To determine whether the addition of digital chest x-ray with Computer Aided Diagnosis (DCXR-CAD) plus FujiLAM screening for all PLHIV at admission to hospital increases the number of participants starting on TB treatment, compared to usual care.	1.1 Proportion of participants being prescribed TB treatment prior to discharge from hospital (including on the day of discharge), or prior to 56 days (whichever is shorter).
**Secondary objective**	
2.1. To determine whether the systemic addition of DCXR-CAD plus FujiLAM to all PLHIV at admission to hospital reduces all cause inpatient mortality, compared to usual care.	2.1. Time (in days) to death from any cause, censored at 56 days from recruitment.
2.2. To determine whether the systemic addition of DCXR-CAD plus FujiLAM screening for all PLHIV at admission to hospital reduces the number of participants with undiagnosed sputum-culture positive TB, compared to usual care.	2.2. Proportion of participants with a sputum culture positive for *M. tb* who were not on TB treatment at the earliest timepoint from;<br>• the time of discharge from hospital<br>• 56 days after recruitment into study the time the culture result is reported.
2.3. To determine whether the systemic addition of DCXR-CAD plus FujiLAM screening for all PLHIV at admission to hospital increases the number of people starting TB treatment on the same	2.3. Proportion of all participants who are started on TB treatment within 24 hours from recruitment into study.

Approved by
College of Medicine
10-Dec-2020
(COMREC)
Research and Ethics Committee

day as diagnostic tests.	
**Other planned analyses**<br><br>To determine the impact of systematic addition of DCXR-CAD plus FujiLAM on all PLHIV at admission to hospital on;<br><br>3.1 Inpatient mortality.<br><br>3.2 56 day mortality (measured as a proportion rather than time to event)<br><br>3.3 The proportion of TB diagnoses that are microbiologically confirmed disease vs. clinically diagnosed.<br><br>To ascertain process outcomes;<br><br>3.4 To determine intervention fidelity.<br><br>To assess the diagnostic accuracy of DCXR-CAD in this population;<br><br>3.5 Sensitivity, specificity, positive and negative predictor value compared to (a) a composite microbiological gold standard and (b) a clinical reference standard.	3.1 Proportion of participants who die prior to discharge from hospital (censored at 56 days).<br>3.2 Proportion of participants who die in 56 days following recruitment.<br>3.3 Proportions of participants started on TB treatment with (a) a microbiologically confirmed diagnosis of TB and (b) clinically diagnosed TB disease<br><br>3.4 Proportion of participants randomised to DCXR-CAD arm who have a valid CXR and CAD score recorded.<br><br>3.5 Sensitivity, specificity, PPV, NPV compared to (a) positive *M. tb* culture from sputum sample or pleural fluid, or positive Xpert MTB/Rif result on sputum or pleural fluid or two or more sputum smears positive for AFBs on microscopy or (b) micrologically confirmed TB **or** a decision to start TB treatment.
**Descriptive or exploratory objectives (enhanced diagnostic cohort arm and those being investigated for HIV virological failure)**<br><br>4.1 To describe the proportion of partcipants meeting a clinical or clinical / microbiological description for<br>Sepsis<br>Invasive bacterial disease<br>Cryptococcal disease<br>Pneumocystis jirovecci pneumonia<br>Bacterial pneumonia<br>Immune reconstitution inflammatory	4.1 Descriptive analysis. See definitions in appendix 1. Participants can have more than one diagnosis (or none). Stratified by CD4 count category.

Approved by College of Medicine
10-Dec-2020
(COMREC) Research and Ethics Committee

syndrome (IRIS)<br>HIV treatment failure<br><br>4.2 To describe the range of values of inflammatory markers and blood TB biomarkers and the relationship between these tests and infectious diagnoses.	4.2 Descriptive analysis.
4.3 To describe the difference in all-cause mortality between the group provided with enhanced diagnostics and those with usual care diagnostics (exploratory outcome).	4.3 Proportion of participants experiencing inpatient mortality in enhanced diagnostic group compared to trial arms.
4.4 To describe the prevelance of HIV virological failure, and ART resistance mutations.	4.4 Descriptive analysis

Approved by
College of Medicine
10-Dec-2020
(COMREC)
Research and Ethics Committee

6. STUDY DESIGN

A pragmatic, single-centre (Zomba Central Hospital, Malawi) cluster randomised, clinical trial with two trial arms and a third observational enhanced diagnostic arm. The observational diagnostic arm will not contribute to trial outcomes. The unit of randomisation is admissions day (see section 8.1). Clusters (days) will be randomised in a 4:4:1 allocation ratio to one of the two study arms or to the enhanced diagnostic cohort. Neither participants nor research staff interacting with participants will be blinded to study arm, but investigators working with data will remain blinded to allocation as far as possible. All adult patients with HIV being admitted to medical wards who are willing and able to consent are eligible for study inclusion, regardless of presenting symptoms or ART status.

Participants randomised to the standard of care arm will receive usual care alone. Usual care includes tests routinely available at Zomba Central Hospital, including (but not limited to) urine Alere LAM and sputum Xpert Mtb/Rif on treating clinician request. Participants randomized to the intervention arm will receive usual care plus urine FujiLAM screening plus a DCXR-CAD, with CAD score and FujLAM results appended into their medical notes. If participants have a CAD score above a pre-determined threshold (see section 9.9) the study team will attempt to collect sputum for Xpert Mtb/Rif. Chest X-ray images will be available for clinicans to view on computers in the ward.

Participants in the observational enhanced diagnostic arm will receive an enhanced package of diagnostics as described in section 9.7 and appendix 2. Participants in the trial arms may optionally also 'opt in' to provide samples for HIV viral failure investigations.

The usual medical team will be responsible for all clinical management decisions, including the decision to start TB treatment. We will recommend that TB treatment is started is FujiLAM screening is positive.

Outcomes will be ascertained at 56 days following recruitment into study.

Approved by
College of Medicine
10-Dec-2020
(COMREC)
Research and Ethics Committee

7. STUDY POPULATION

7.1. Trial Participants

Adults (aged ≥18 years) living with HIV infection (PLHIV) who require acute admission to medical wards at Zomba Central Hospital and who are willing and able to provide informed consent will be enrolled, regardless of clinical presentation and whether or not TB is clinically suspected.

7.2. Inclusion Criteria

All medical admissions will be screened for eligibility, regardless of the presenting complaint.

Inclusion criteria are;

- Requires acute admission to a hospital medical ward at Zomba Central Hospital for any reason
- Have HIV-infection (existing or new diagnosis, irrespective of ART status)
- Willing and able to give informed consent

7.3. Exclusion Criteria

- Aged <18 years
- Have been admitted to a medical ward for longer than 18 hours
- Taking TB treatment before admission or has received treatment for TB within the preceding 6 months.
- Have already been in the study during an earlier hospital admission.

Approved by
College of Medicine
10-Dec-2020
(COMREC)
Research and Ethics Committee

8. RECRUITMENT, RANDOMISATION AND BLINDING

8.1. Randomisation, allocation and masking of clusters (days)

A randomisation list will be generated in advance with random block size to ensure equal numbers of days are enrolled into each arm. Allocations of clusters will be printed and placed into sequentially numbered opaque sealed envelopes. One envelope will be open each day prior to study recruitment commencing to reveal the allocation for that cluster (day).

None of the Zomba-based research teams, usual care medical team or participants will be blinded to trial arm allocation.

However, in the course of managing data, investigators will remain masked to allocation as far as possible until database lock preceding final analysis. Data monitoring and data cleaning will be done without reference to group allocation. No unblinded interim analysis will be conducted, unless requested by the DSMC. Should the DSMC request an unblinded interim analysis, a statistician from Malawi-Liverpool-Wellcome will run the statistical code to produce unblinded analysis and only the MLW statistician, Prof Fielding (who will represent the trial in a closed DSMC session) and the DSMC will see the unblinded results – the Principal Investigator and other co-investigators will remain blinded.

8.2 Screening and recruitment of participants

All adult PLHIV admitted to medical wards (irrespective of clinical presentation and the reason for medical admission) will be referred by health service staff or approached directly by the study team for explanation of the study and assessment of eligibility. All adults of unknown HIV status are offered HIV testing (provider-initiated testing and counseling, PITC) on admission as standard clinical practice. If potential participants report being HIV positive, confirmation will be sought either through confirmation documentation (e.g. health passport or inspection of ART medications) or confirmatory HIV testing offered by the study team.

Information about the study given to patients by study team when inviting for study enrollment screening will be the same regardless of which trial arm is being recruited on that day.

Patients will be screened and offered recruitment to the study until 3pm in the afternoon. The late afternoon cut off is to allow time for study recruitment procedures to take place and to have a FujiLAM urine screen, chest x-ray, CAD score and Xpert (if indicated, and sputum production possible) completed on the day of recruitment.

Patients who attend Zomba in the late afternoon or evening after the cut-off time will not be recruited on that day. However patients who attend in the evening and who are admitted, will be eligible for recruitment on the following day.

Those willing to be screened for eligibility will be assigned a screening ID number and assessed by the study team as per the above criteria. Screening and recruitment logs will be kept for all potentially eligible patients admitted to each study site and screened patients who either are or are not enrolled, including the reasons for non-enrollment when available.

Approved by
College of Medicine
10-Dec-2020
(COMREC)
Research and Ethics Committee

8.3 Informed Consent

Written informed consent will be sought from participants before any trial-specific procedures are undertaken, with witnessed thumbprint used for participants unable to read and/or write. A copy of the participant information leaflet and signed informed consent will be given to the participant. It will be clearly stated that the participant is free to withdraw from the trial at any time for any reason without prejudice to future care, and with no obligation to give the reason for withdrawal.

Approved by
College of Medicine
10-Dec-2020
(COMREC)
Research and Ethics Committee

9. INTERVENTIONS, METHODS AND PROCEDURES

9.1 Baseline Assessments and Procedures (trial arms)

Following enrolment the study team will collect information on:

- Age and sex
- Time and date of admission to hospital
- Whether TB was an admitting differential diagnosis
- WHO four symptom TB screening (presence of cough any duration, night sweats, fever or weight loss)
- Whether the participant is currently on ART
- Whether the participant is able to walk unaided
- Address information and mobile phone numbers of the participant and/or designated relatives/next-of-kin.

The study team will be responsible for obtaining, where possible:

- One research sputum specimen (spontaneously expectorated) for mycobacterial culture.
    - If participants are unable to produce a sputum sample initially, they will be instructed on how to do so and left with a sputum specimen container - the research team will periodically attempt again to collect sputum for culture over the course of admission.

If participants choose to opt in to study of HIV virological failure we will also

- Collect information on ART regimen and adherence
- Obtain a 12mL (three teaspoon) blood sample (8mL lithum heparin tube, 4mL purple top tube)

When participants are recruited on intervention days the study team will also arrange, where possible:

- A urine sample (7mL) to be tested with FujiLAM and AlereLAM
- A digital chest x-ray image with CAD score.
- If the CAD score is above a predetermined threshold (determined in a pilot study, see 9.9) then the study team will ask the participant for a sputum sample (in addition to the study sample collected for culture) for Xpert MTB/Rif.
- CAD score and interpretation ("TB likely" vs. "TB not likely") will be appended into participant's notes.

9.2 Baseline assessments and procedures (enhanced diagnostic observational arm)

In addition to sputum samples and chest x-ray (as detailed above in section 9.1), for participants in the enhanced diagnostic arm only, the research clinical officer will be responsible for obtaining, where possible;

- 22mL blood (5mL for blood culture, 6mL in EDTA blood tube, 4mL in a plain tube and 8mL in a lithium heparin tube).

Approved by College of Medicine 10-Dec-2020 (COMREC) Research and Ethics Committee

In addition to the baseline questions for the trial arm groups, the study team will also collect information on;

- Presenting complaint
- Systematic review of symptoms
- Vital signs, including SpO2 measurement
- Karnofsky score
- Past or current TB treatment, HIV care, ART duration.
- Validated ART compliance score (where applicable). [28]
- Address information and mobile phone numbers of the participant and/or designated relatives/next-of-kin

9.3 TB Definitions

TB events will be defined as follows for the purpose of this study:

Microbiological diagnosis of TB (confirmed); will have at least two positive Acid Fast Bacilli (AFB) smears or one or more Xpert Mtb/Rif positive or one or more culture positive for *M. tb* on any specimen or a positive urine LAM result. One study sputum culture will be collected per participant, TB results will also be collected from any other samples organised by the routine health services.

Microbiological diagnosis of TB (probable); will have one positive AFB smear from any site.

Microbiological diagnosis of pulmonary TB (confirmed); will have at least one smear-microscopy, Xpert and/or culture positive result(s) on a sputum sample or pleural fluid sample, whether from study sample or routine care sample.

Microbiological diagnosis of pulmonary TB (probable); will have one positive AFB smear from a sputum sample.

Clinical diagnosis of TB; will have a compatible clinical illness and the decision of the responsible clinical team to commence TB treatment in the absence of any positive microbiological tests for TB.

Undiagnosed TB; refers specifically to participants who do not have a microbiological diagnosis of TB made on the basis of study or usual care samples, and have not been empirically started on TB treatment following a clinical diagnosis of TB, and have culture-positive *M. tb* on study sputum culture.

Chest X ray suggestive of TB; will be a CAD score about a pre-determined threshold. The threshold will be determined in the piloting phase (see section 9.9).

Date of TB diagnosis; for microbiological TB diagnoses this will be the date when the positive microbiological test result for TB was communicated to the responsible medical team (eg written in the hospital record), and for clinical diagnoses it will be the date of commencement of TB treatment.

Date of commencement of TB treatment; will be the first date that the participant was recorded in the TB register as having initiated TB treatment.

Time of commencement of TB treatment; will be the time that TB treatment for the specific participant is dispensed from pharmacy to the participant (or their guardian or a ward nurse).

Approved by
College of Medicine
10-Dec-2020
(COMREC)
Research and Ethics Committee

9.4 Definitions of clinical / microbiological diagnoses (enhanced diagnostic arm only)

See appendix 2

9.5 Training for responsible medical team

In addition to training in the trial protocols and procedures, staff responsible for the routine delivery of care for patients who are participants in this trial will be educated with regards to use of chest x-ray for diagnosis of TB in general and the interpretation of CAD scores in particular. This will include information about the sensitivity and specificity of chest x-ray and CAD and estimated setting-specific positive and negative predictive values for TB diagnosis, allowing them to make informed decisions on how to interpret test results.

The study will also provide training on the interpretation of FujiLAM, particularly that it is expected to be more sensitive than Alere LAM.

9.6 Subsequent procedures (trial arms)

Follow-up during hospital admission

The study team will have no role in routine patient investigation, care and management. Participants will remain under the care of their responsible medical teams who will be responsible for all clinical decisions including initiation of TB treatment, ART, co-trimoxazole prophylaxis and treatment for co-morbidities according to local and national guidelines. We will recommend that the usual team commence TB treatment is FujiLAM is positive.

The study team will extract records of during hospital admission by regular review of clinical records and/or discussion with medical team. Information will be recorded in a CRF about events during admission;

- Results of TB tests organized by the routine team
- Commencement of TB treatment, date and time of starting treatment, reasons for commencement (e.g. microbiological results, chest x-ray or clinical symptoms in the absence of positive microbiological tests).
- Vital status whilst in hospital, including date of death
- Date of discharge and length of hospital stay
- Serious or severe adverse events (see section 11)

Sputum culture results

For participants able to produce sputum, a single sample will be sent for mycobacterial culture and identification at the COM/MLW TB Reference Laboratory in Blantyre, Malawi.

The study team will be informed about positive culture results. If the participant is still an inpatient the report (positive or negative) will be communicated by the study team to the responsible medical team and written in their medical notes. For participants who have been discharged results will be reported to the district TB officer. If a participant has a positive result and was not commenced on TB treatment the result will also be reported to the participant themselves - the study team will attempt to contact the participant via mobile phone and via relative's mobile phone if necessary.

Approved by
College of Medicine
10-Dec-2020
(COMREC)
Research and Ethics Committee

Follow up at 56 days from recruitment

At 56 days from recruitment, the study team will attempt to contact participant via mobile phone. If they cannot reach the participant by phone they will call telephone numbers of relatives provided by the participant at recruitment. If there is still no response, home tracing will be attempted. The purpose is to ascertain vital status (alive or dead) only, with no other follow up questions. Follow up attempts will start at 56 days from recruitment and attempts will continue to be made for up to 14 more days beyond this time.

If the participant opted in to have tests for HIV virological failure**, and** had HIV virological failure at recruitment (HIV virus detected) then we will recall them for an in-person visit at 56 days for a further blood sample to see if they have re-supressed their viral load. At this visit we will take a further 12mL blood sample (8mL lithium heparin tube and 4mL purple tube) to repeat tests of HIV viral load and viral resistance.

9.7 Subsequent procedures (enhanced diagnostic observational arm only)

Follow-up during hospital admission

The study team will extract records of TB treatment initiations during hospital admission by regular review of clinical records, the TB register, and discussion with medical team.

Information will be collected about events during admission;

- Any TB tests organized by the routine team, including nature of specimens and results
- Any other laboratory test organized by the medical team.
- TB diagnosis, including date of diagnosis.
- Commencement of TB treatment, date of starting treatment, reasons for commencement (e.g. microbiological results, chest x-ray or clinical symptoms in the absence of positive microbiological tests).
- Initiation or administration of ART, including regimen and timing.
- Clinical response to ART to detect any syndrome compatible with IRIS based on ACTG clinical definition (see appendix 2). [29]
- Initiation or administration of co-trimoxazole therapy.
- Initiation of isoniazid preventative therapy,
- Concomitant bacterial infection or prescription of antimicrobials (other than TB treatment).
- Other opportunistic infections based on tests or treatment given.
- Vital status, including time of death.
- Time to discharge and length of hospital stay.
- Serious or severe adverse events (see section 11).

TB specimens and results:

In addition to the sputum for culture at MLW / COM laboratory in Blantyre, a further sputum sample will be submitted to the hospital laboratory for Xpert MTB/RIF testing. Results of Xpert tests will be issued to the responsible medical team as soon as available and communicated to the responsible medical team to inform decisions regarding TB treatment (anticipated to be within 24-48 hours of admission).

Results from sputum culture will be reported in the same way as participants in the two main trial

Approved by
College of Medicine
10-Dec-2020
(COMREC)
Research and Ethics Committee

arms (see above, section 9.6)

Other specimens and results:
Other test results (CD4 count, haemoglobin, HIV viral load if indicated) will be communicated to the responsible medical team as soon as results are available. Where necessary, clinical advice about interpretation of blood culture results and antibiotic sensitivities will be provided with laboratory results.

Some results will be processed in batch (inflammatory markers, TB biomarkers) retrospectively and these results will not be available in a "real time" fashion to influence clinical care. Results for some samples (HIV drug resistance testing and HIV drug levels) will require samples to be transported outside of Malawi, under the terms of a Material Transfer Agreement (MTA). Participants will be asked to give their express consent for this shipping in the ICF.

The routine investigation and management of partcipants according to local protocols will not be altered. Participation in the trial will not prevent the responsible medical team from requesting additional TB (or other) diagnostic tests if clinically indicated.

Follow up at 56 days from recruitment
At eight weeks from recruitment, the study team will contact participant via mobile phone. If they cannot reach the participant by phone they will call numbers of relatives provided by the participant at recruitment. If there is still no response, home tracing will be attempted. The purpose is to ascertain vital status (alive or dead) only with no other follow up questions. Follow up attempts will start at 56 days from recruitment and attempts will continue to be made for up to 14 more days beyond this time.

If the participant had HIV virological failure at recruitment (HIV virus detected) then we will recall them for an in-person visit at 56 days for a further blood sample to see if they have re-supressed their viral load.

9.8 Storage of specimens

No samples will be stored from participants in either of the two trial arms unless they opt in to HIV virological failure detection. In this case case plasma samples will be stored as below.

For participants in the enhanced diagnostic arm or who opt in to HIV virological failure detection, plasma samples and sputum samples will be stored for up to five years for planned studies on TB biomarkers. Participants will be asked to give their express consent for storage of samples for future research on TB diagnostic tests.

9.9 Pilot phase procedures

Prior to trial recruitment, the study will be preceded by a 4-8-week piloting phase to identifying any issues that require resolution, to finalise standard operating procedures, and to ensure data-collection systems are robust. No participants will be randomly allocated to interventions during this period.

Activities to be undertaken during the pilot phase will include:

Approved by
College of Medicine
10-Dec-2020
(COMREC)
Research and Ethics Committee

- Measurement of rates of hospital attendance and prevalence of TB.
- Assessment of timing of participant presentation throughout the working day in order to determine a time cut off for daily recruitment.
- Piloting of study questionnaire completion.
- Piloting of participant physical flow through admission unit and radiography room.
- Piloting of digital chest x-ray and CAD system, including defining a CAD threshold score.
- Piloting of sputum collection procedures.
- Piloting of urine collection procedures
- Piloting of laboratory procedures
- Establishment and refinement of quality control procedures.

During this phase participants will be identified and screened according to the protocol above, but with no randomisation. We anticipate recruiting 40 – 50 participants in the pilot phase.

Participants who consent to be in the study during the pilot phase will undergo a chest x-ray with CAD score and have sputum samples collected for both mycobacterial culture in Blantyre and GeneXpert MTB/Rif locally.

9.10 Study timeline

The timeline proposed for the study is below.

	**2019**					**2020**												**2021**												**202**
	**A**	**S**	**O**	**N**	**D**	**J**	**F**	**M**	**A**	**M**	**J**	**J**	**A**	**S**	**O**	**N**	**D**	**J**	**F**	**M**	**A**	**M**	**J**	**J**	**A**	**S**	**O**	**N**	**D**	**j**
Ethical approval and permissions	X	X																												
Recruit study team and study staff training.			X	X	X																									
Write SOPs and investigator file		X	X	X																										
Piloting phase						X	X																							
Participant recruitment													X	X	X	X	X	X	X	X	X	X	X	X	X	X				
Follow-up participants														X	X	X	X	X	X	X	X	X	X	X	X	X	X	X		
Database lock and data analysis																												X	X	
Write up and disseminate results.																												X	X	X

Approved by
College of Medicine
10-Dec-2020
(COMREC)
Research and Ethics Committee

10. OUTCOME EVALUATION

10.1 Follow-up procedures and ascertainment of outcomes (trial arms)

Participants will be contacted by the study team by telephone at 56 days to ascertain vital status. If the study team is unable to contact the participant they will contact the next of kin via telephone. If there is no response to several telephone calls on several days, the study team will attempt a home visit.

The usual medical team will arrange follow up (including HIV and ART care) according to usual practice.

10.2 Follow-up procedures and ascertainment of outcomes (enhanced diagnostic observational arm)

Participants enrolled in the enhanced diagnostic observational arm will be contacted by the study team by telephone at 56 days to ascertain vital status. If the study team is unable to contact them, they will contact the next of kin via telephone. If there is no response to several telephone calls on several days, the study team will attempt a home visit

The usual medical team will arrange clinical follow up (including HIV and ART care) according to usual practice.

10.3 Discontinuation/Withdrawal of Participants

Each participant has the right to withdraw from the trial at any time. The reason for withdrawal (if the participant wishes to give a reason) will be recorded in the study termination CRF.

10.4 Definition of End of Trial

The trial will be considered closed following the completion of follow up of the last participant, and once all follow-up and laboratory reports have been received.

Approved by
College of Medicine
10-Dec-2020
(COMREC)
Research and Ethics Committee

11. SAFETY REPORTING

The main study-related procedures in this trial are systematic screening of hospitalised PLHIV for TB using DCXR-CAD and urine FujiLAM, in addition to usual care TB screening and clinical assessment. Usual care TB screening for all PLHIV being admitted to hospital Malawi includes urine Alere LAM and sputum Xpert when requested by clinicians.

It will not be possible to distinguish true-positive CAD results from false-positive CAD results on an individual basis, owing to the lack of a highly sensitive and specific gold-standard confirmatory TB test. However, given the high mortality from undiagnosed TB in this patient group, combined with the high frequency with which empirical TB treatment is used in routine care, and the established good safety profile of standard tuberculosis treatment, the overall potential for harm to participants in the intervention arm is low. WHO recommend chest x-ray in severely-ill PLHIV. TB treatment initiation on clinical grounds alone (with or without chest x-ray evidence of TB) in people who are unwell and may have TB is in keeping with WHO guidance (WHO algorithm in section 4.1, and see also section 14.3).

A single chest x-ray typically delivers an average effective radiation dose of 0.01mSv, comparable to 10 days of natural background radiation or 30 minutes on a commercial aeroplane flight, and with less than one in a million chance of causing cancer. The potential benefits of chest x-ray as a TB diagnostic tool are likely to outweigh this very small risk, and this is in keeping with current WHO recommendations. [9 10]

Standard Serious Adverse Event (SAE) reporting will not be possible or appropriate for the reasons outlined above, the trial team will instead investigate and report periodically to the TSC, DSMB and Ethics Committees all instances of

a. Erroneous reporting of laboratory results leading to a participant starting TB therapy in error
b. Breach of confidentiality following TB or HIV diagnosis
c. Needlestick injuries

Deaths will also be systemically recorded as study outcomes and reported to the DMSB and TSC periodically.

Approved by
College of Medicine
10-Dec-2020
(COMREC)
Research and Ethics Committee

12. STATISTICS AND SAMPLE SIZE JUSTIFICATION

12.1 Assumptions

In STAMP the observed morality in Malawi was 11.3% as an inpatient and 22% by 56 days. This is lower than other mortality estimates from cohorts of PLHIV inpatients in the WHO AFRO region. [1,21,22] In our pilot phase the inpatient mortality point estimate was slightly lower (but with a very wide confidence interval).

We observed that 14% of all patients were started on TB treatment in STAMP Malawi arm, (18% of patients in Alere LAM arm and 10% of patients in sputum diagnostics only arm). Of note, in STAMP empiric TB treatment was less common in Malawi (2% of all patients / 11% of all TB treatment initiations in Alere LAM arm) than in South African (9% of all patients / 40% of all TB treatment initiation in Alere LAM arm). [2] In our pilot phase TB treatment initiations were closer to that seen in the usual care arm of STAMP rather than the LAM arm (again, with a very wide confidence interval).

There are no previous studies to provide robust effect size estimates for either of our primary outcomes.

The STAMP trial showed an increase in TB treatment and a non-significant overall reduction in mortality at 56 days when Alere LAM was introduced for TB screening from 21% to 18% (mortality reduction was statistically significant in predefined subgroups of those with CD4 count <100 cells/µL, haemoglobin <8g/dL and where TB was in the differential diagnosis at admission). [2]

We assume a cluster size of 3 - 4 (ie. 3 - 4 people living with HIV admitted per day) based on routine data from Zomba hospital. We assume clusters are relatively similar to each other with a ρ of 0.005. A similar day-of-the-week randomisation design of partner HIV testing among antenatal outpatients in Malawi showed a ρ (0.015). [30]

12.2 Sample size and power (primary outcome)

We assume TB treatment initiation in the control group of 10% (based on data from pilot phase) and our hypothesis is that the DCXR-CAD plus FujiLAM intervention will increase this to 18% (i.e. an absolute risk difference of 8%).

12.3 Sample size and power (secondary outcomes)

We assume 20% of participants in usual care arm will experience death by 56 days (i.e. survival probability 0.8) and hypothesise a hazard ratio of between 0.5 and 0.8.

We assume 1% of participants in usual care arm will start TB treatment within 24 hours (this was not observed at all in our pilot phase) and hypothesise this intervention could increase this to 5%.

We assume 10% of participants have undiagnosed TB – based on the relatively low rate of TB treatment initiation observed in pilot (there are no sputum culture results available from the pilot as of the date of this protocol revision) and hypothesise that this intervention could reduce this to 5%.

Approved by
College of Medicine
10-Dec-2020
(COMREC)
Research and Ethics Committee

As above, for all secondary outcomes we assume cluster size 3 - 4, rho 0.005 and co-efficient variation of cluster size 0.05.

The below table shows the study power and sample size calculations. Appendix 3 contains graphs showing the effect on cluster size and power as assumptions are altered.

**Primary outcome (1): Proportion of people started on TB treatment by discharge from hospital**									
Cluster size	Proportion started on TB treatment (usual care arm)	Risk difference (increase)	CV cluster size	ρ	1-β	α	Clusters / trial arm	Participants / trial arm	Weeks recruitment (inc. diagnostic cohort clusters)
**3**	0.1	**0.06**	0.05	0.005	0.8	0.05	166	498	94
**3**	0.1	**0.08**	0.05	0.005	0.8	0.05	100	300	57
**4**	0.1	**0.06**	0.05	0.005	0.8	0.05	75	300	43
**4**	0.1	**0.08**	0.05	0.005	0.8	0.05	126	504	71

**Secondary outcome (1): Hazard of death by 56 days**							
Cluster size	Survival probability (usual carel arm)	Hazard ratio	CV cluster size	ρ	α	Clusters per trial arm	Power (%)
**3**	0.2	**0.6**	0.05	0.005	0.05	102	69%
**3**	0.2	**0.8**	0.05	0.005	0.05	102	21%
**4**	0.2	**0.6**	0.05	0.005	0.05	102	82%
**4**	0.2	**0.8**	0.05	0.005	0.05	102	26%
**Secondary outcome (2): Proportion of TB treatment initiations within 24 hours from recruitment.**							
Cluster size	Proportion all participant on TB treatment 24 hours (usual care arm)	Risk difference (increase)	CV cluster size	ρ	α	Clusters per trial arm	Power (%)
**3**	0.01	0.05	0.05	0.005	0.05	102	82%
**4**	0.01	0.05	0.05	0.005	0.05	102	91%
**Secondary outcome (3): Prevalance of undiagnosed TB as discharge**							
**3**	0.1	0.05	0.05	0.005	0.05	102	64%
**4**	0.1	0.05	0.05	0.005	0.05	102	76%

Table 1: Power and sample size calculations under a variety of different effect size, cluster size and cluster similarity values.

We aim to recruit 102 clusters per trial arm, with approximately 306 participants per arm. A further 26 clusters will be recruited in the enhanced diagnostic observational arm, with approximately 78 participants (these clusters do not contribute to trial outcomes). Total study size will therefore be 690 people, recruited in 230 clusters (days).

This will give 81% power with a 5% type 1 error rate to detect an absolute increase of 8% (from a baseline of 10%) TB treatment initiations.

12.4 Data analysis plan

A detailed statistical analysis plan will be written and approved by the DSMC prior to database lock and unblinding. Trial reporting will follow CONSORT Guidelines.

We will report baseline characteristics of randomised participants, stratified by allocated group.

Approved by
College of Medicine
30-Dec-2020
(COMREC)
Research and Ethics Committee

Analysis of the primary and secondary outcomes will be done on an intention to treat basis, with all participants allocated to trial groups included and analysed in the group to which they were randomized (regardless of which intervention was received). Adjustment to statistical estimates will be made (using random effects model) to take into account clustering of outcome by days in this cluster-randomised trial design.

Trial analyses of proportion of patients started on TB treatment (primary outcome) will be compared using risk differences and odds ratios.

Trial analyses of time from randomization to all-cause mortality (secondary outcome) will be compared between intervention arm and usual care arm using survival analysis with Kaplan Meier curves and Cox proportional hazards regression, with the intervention effects summarised by a hazard ratio. Outcomes will be censored at 56 days from recruitment.

Proportions of participants starting TB treatment within 24 hours from recruitment and proportion with undiagnosed TB will be analysed as a secondary endpoints using risk difference and odds ratios.

Descriptive statistics (proportions, means and medians as appropriate) will be used to describe the characteristics of participants and their diagnoses in the diagnostic cohort arm. As a planned analysis the time from randomisation to death will be compared between participants in the enhanced diagnostic cohort arm and the trial intervention arm using survival analysis with Cox proportional hazards; this is an exploratory analysis and the study is not powered for this.

Approved by
College of Medicine
10-Dec-2020
(COMREC)
Research and Ethics Committee

13. DATA MANAGEMENT

13.1 Data Recording and Record Keeping

Electronic case report forms (CRFs) will be completed for each participant as follows;

- Eligibility and screening (collected from participant and medical records)
- Baseline demographic and clinical information (collected from participants where possible, supplemented by information from hospital records)
- Laboratory test results (collected from hospital and laboratory records)
- In-patient hospital follow-up (data collected from hospital records and/or clinical team)
- Hospital discharge or in-patient death (data collected from hospital records and/or clinical team)
- Out-patient follow-up, loss-to-follow-up or outpatient death (data from next-of-kin, relative or official register).

CRFs will be completed electronically on tablets using forms developed in Open Data Kit, preprogrammed with logical data consistency checks. Encrypted data files will be sent via secure internet connection to the study data-hub at the Malawi-Liverpool-Wellcome Trust. Xray images files will be held on secure encrypted password-protected computers and on a secure encrypted server.

The PI will monitor data-completeness and quality and generate lists of data queries on a weekly basis which will be issued to the Zomba-based study team for review, clarification and feedback. All electronic databases will have daily scheduled backups, and a password will be required to gain access to data. The password-protected databases at the trial site will be sent to LSHTM by Secure File Transfer Protocol (SFTP). Only trained staff will be granted access on a need-to-see basis.

Data will be stored using a unique study identifier allocated to each participant at enrolment into the study. Participant name and any other identifying detail will not be included in the main trial data electronic file. In accordance with data protection regulations, identifiers will be deleted entirely from the database as soon as practical to render it fully anonymous.

Paper consent forms will be transported from Zomba to MLW and stored securely.

Direct access will be granted to authorised representatives from the sponsor, host institution and the regulatory authorities to permit trial-related monitoring, audits and inspections.

All staff will be trained with respect to data management issues and GCP.

Approved by
College of Medicine
10-Dec-2020
(COMREC)
Research and Ethics Committee

14. MONITORING AND QUALITY ASSURANCE

The trial will be conducted in accordance with the current approved protocol. The investigators will ensure that this trial is conducted in accordance with the principles of the Declaration of Helsinki, with the ICH Guidelines for Good Clinical Practice (CPMP/ICH/135/95) July 1996, MRC Guidelines for GCP, and relevant regulations and standard operating procedures (SOPs).

Data quality checks will be inbuilt to the data recording systems. In addition, all data collected will be subject to random sampling for verification of accuracy in relation to source documents. Any problems with data quality will be reported to the PI and appropriate action taken, including increasing frequency of checks.

The London School of Hygiene & Tropical Medicine (LSHTM) will act as the main sponsor for the study and the study may be subject to audit by LSHTM under their remit as sponsor, or assessment by the regulatory authorities, to ensure compliance with protocols, GCP and applicable regulatory requirements.

14.1 Data Safety Monitoring Committee

The Data Safety monitoring committee (DSMC) will monitor progress, advise the PI and investigator team, review safety data and report to the trial funders and sponsor. The DSMC will include an independent chairperson, principal investigator, co-investigators, trial statistician and independent experts. The TMC will be established comprising three independent members. The DSMC will meet prior to trial start and make recommendations concerning adverse events.

14.2 Trial Investigator Team

The investigational team draws on experience of a very strong team of local and international partners with a wealth of clinical experience in diagnosis and management of HIV-associated TB; research experience in clinical evaluation of TB diagnostics; design and contduct of randomized controlled trials; data management and analysis and strong relationships with local, regional and national partners such as TB programmes.

- Dr Rachael Burke (Clinical PhD fellow): Principal Investigator
- Prof Liz Corbett (Professor of Tropical Epidemiology): PhD supervisor and MLW TB / HIV group lead.
- Dr Peter MacPherson (Reader and Wellcome Trust Fellow): PhD supervisor and MLW Public Health Group Lead.
- Dr Ankur Gupta-Wright (Academic Clinical Lecturer): PhD supervisor
- Prof Katherine Fielding (Professor of Medical Statistics & Epidemiology and Director of LSHTM TB Centre): PhD advisor and co-investigator
- Dr Naomi Walker (Liverpool School of Tropical Medicine): PhD advisor and co-investigator
- Dr Marriot Nliwasa (College of Medicine): co-investigator
- Dr Saulos Nyirenda (Zomba Central Hospital): co-investigator
- Dr James Mpunga (National TB Programme): collaborator
- Rose Nyirenda (Department HIV / AIDS): co-investigator
- James Kandulu (Department diagnostics, Ministry of Health): collaborator

Approved by
College of Medicine
10-Dec-2020
(COMREC)
Research and Ethics Committee

14.3. Ethical Considerations and Approvals

The protocol, informed consent form and participant information sheet will be submitted to Research Ethics Committees (REC) of the London School of Hygiene & Tropical Medicine and the College of Medicine Research and Ethics Committee (COMREC).

The Investigator will submit and, where necessary, obtain approval from the above parties for all substantial amendments to the original approved documents.

Reporting:
The investigators shall submit an annual progress report to the COMREC and LSHTM REC once a year throughout the clinical trial. In addition, an End of Trial notification and final report will be submitted to the REC and host organization.

Expenses and Benefits
All participants are hospital inpatients and will not incur additional transport costs or lost time from economic activities due to participation in the study. [31]

Participants in the enhanced diagnostic arm will be compensated for their time and inconvenience of sample collection. Approximately 30 minutes of time whilst in hospital will be asked of participants to respond medical questionnaire delivered by a research assistant, in addition to screening questions. Participants will be asked to provide 22mL of blood (in a single blood draw) and urine sample and two sputum samples. Participants will be reimbursed equivalent of US$10 in MKw.

Participants in the trial arms will only be required to complete study screening questions and provide very minimal study information (age, sex, 4 TB symptom questions, ever had ART and ascertainment of whether they can stand). A single sputum and a urine sample will be requested, but no other study samples will be collected. Participants admitted on days randomised to the intervention arm will have a DCXR. Participants will be reimbursed the equivalent of US$1 in MKw.

Risks to patients

Risk of harm to participants in this trial is low.

Chest x-ray for all PLHIV who are seriously unwell is already international best practice, and endorsed by WHO, although currently many hospitals in Malawi don't have routine availability of chest x-ray. WHO has recommended that *"Computer aided diagnosis can be used for TB detection for research, ideally following a protocol that contributes to the required evidence base for guideline development."* Therefore, care being provided in the intervention arm is in keeping with international best practice and care in the control arm is in line with national standards and thus risk to participants is low. No drug treatment is mandated as part of the study, and all medicines that participants will likely receive from their usual team as a result of participating in the study are licensed drugs being used for their licensed indications.

FujiLAM is a CE-marked in vitro diagnostic test designed for the purpose of TB diagnosis.

Approved by
College of Medicine
10-Dec-2020
(COMREC)
Research and Ethics Committee

The main potential harms specifically attributable to the intervention arm of the trial relate to more intensive use of chest x-ray as a diagnostic test and unwarranted initiation of empiric TB treatment as a result of a false-positive CAD result.

We anticipate that participants in the intervention arm will benefit from DCXR-CAD screening due to acceleration of TB diagnosis. However, it is possible that such screening may inadvertently be harmful in the following ways:

- Whilst a chest X ray can only provide supportive evidence of TB, rather than microbiological confirmation, more people in the Chest X ray arm may receive empiric TB treatment which may include patients who don't have TB.
- Rapid TB diagnosis may reduce the likelihood that participants receive an empirical course of simple antibiotics as part of the diagnostic work-up and therefore concurrent sepsis, if present, may not be treated
- Rapid TB diagnosis may result in other concurrent pathologies being overlooked.

Risks of inappropriate initiation of empiric TB treatment will be minimised by providing refresher training about chest x-ray for diagnosis of TB and the CAD software, clearly communicating the limitations of radiographic screening approaches. The strong recommendation is to attempt microbiological confirmation (Xpert MTB/Rif) in all people with a chest x-ray flagged as "TB likely".

The risks associated with radiation exposure from a single diagnostic chest X ray are extremely low (equivalent to 30 minute aeroplane flight or 10 days background radiation). As discussed above, chest x-ray in this group of patients is already international best practice and as such there is no radiation exposure for study participants beyond that already recommended by international guidelines.

Participant Confidentiality

Generation or perpetration of stigma is a concern when HIV and TB testing and treatment interventions are being offered, although our previous trials and studies suggest that stigmatisation is relatively uncommon and very rarely results in harm or adverse outcomes.

We will take extensive actions to ensure any potential for stigmatization is removed through ensuring that HIV testing and TB screening activities are undertaken in private areas and confidentiality is maintained. The participants will be identified only by unique study identifier on the main electronic database, with identifiers held in a separate password protected electronic database. The trial will comply with the Data Protection Act, which requires data to be fully anonymised as soon as it is practical to do so.

All documents will be stored securely and only accessible by trial staff and authorised personnel. All staff will be trained in the importance of confidentiality and appropriate data handling.

Any breaches of confidentiality will be systematically reported as an adverse event.

Approved by
College of Medicine
10-Dec-2020
(COMREC)
Research and Ethics Committee

14.4 Possible constraints

We do not envisage any major constraints.

The main possible constraint is slower than expected recruitment. We anticipate recruitment within 13 months, but if recruitment is slower than expected, it is possible to extend duration up to 15 months. The study flow chart, salary budget and clinical trial insurance timeframe have been designed to allow for this extension should it be necessary.

Should there be stock-outs of important items for usual care diagnostics (for example, HIV tests) at Zomba hospital, the trial team would work with the Zomba management committee to help resolve the issue. If necessary, the trial may be able to provide funding to source small quantities of routine care diagnostics if needed to bridge a stock-out. Should there be a prolonged power outage the trial team will work with Zomba management committee to assist to resolve the issue if possible.

Other constraints outside of the control of the investigators include local or regional major incidents, for example related to flooding. As long as Zomba Central Hospital is running a medical inpatient service, the trial should be able to continue. Should a major incident occur such that Zomba hospital was required to cease admitting new medical inpatients the trial would be paused until such time as Zomba hospital can re-commence provision of medical inpatient services.

The COVID-19 pandemic has led to a delay of five months in commencing the trial. We have undertaken safety and risk assessments and are planning to resume study activities in August 2020, with appropriate PPE for staff. We have sought an extension from Wellcome Trust due to this delay.

Approved by
College of Medicine
10-Dec-2020
(COMREC)
Research and Ethics Committee

15. BUDGET AND INSURANCE

Funding

The trial is funded by a Wellcome Trust Clinical PhD fellowship award, held by Dr Rachael Burke.

Insurance

The London School of Hygiene and Tropical Medicine (LSHTM) will act as the main sponsor for the study and holds public liability and clinical trial insurance policies which apply to this study which would operate in the event of any participant suffering harm as a result of their involvement in the research.

Requirements and budget justification

The requirements for the trial (together with their costs) are itemised in the budget below.

Personnel include a research clinical officer and two research assistants (field worker grade) who will be responsible for recruitment of participants, completing questionnaires and ascertaining outcomes (from review of ledgers, hospital records and phonecalls to participants). A contribution to the Zomba radiographer and laboratory technician is also included.

Consumables required include sputum mycobacterial culture (which will be done in College of Medicine laboratories in Blantyre) and a contribution towards routine ministry of health provision of usual care tests (HIV tests, Xpert cartridges, LAM strips, CrAg strips). A license fee for the computer aided diagnosis system is also required. Some more specialist tests (for example blood culture) are also required. Consumables related to collecting samples (needles, blood bottles, sputum pots etc.) are budgeted for.

Transport costs are included for transport between Blantyre and Zomba for samples and the study investigator.

Equipment costs are included for costs of a computer, tablets for electronic data collection, refurbishment of the radiography suite at Zomba and an inverter battery for the X-ray unit and costs of a digital X-ray unit.

There are costs related to clinical trial services, mainly incurred at MLW. This includes data management, printing, clinical research support unit time and health and safety. Clinical trial insurance (from LSHTM) is also required and included in the budget.

The budget also includes costs related to PhD training for the principal investigator (this study is funded by her PhD training fellowship). These include PhD fees, travel to London for PhD upgrading, costs for educational modules and costs related to travelling to scientific conferences.

Other requirements not incurring specific costs include desk space at MLW for the principal investigator and space for the study team at Zomba Central hospital to conduct research activities and to store study equipment.

Approved by
College of Medicine
10-Dec-2020
(COMREC)
Research and Ethics Committee

15. PUBLICATION POLICY

Academic dissemination

Data will be disseminated to COMREC, local institutions, academic bodies and professional associations within Malawi with which the members of the investigational team already have links. Data will also be rapidly made available through presentations at relevant leading international conferences and regional conferences. Data will be published in a timely manner in peer reviewed journals. Findings will be summarised and made readily accessible on the institutional web-sites of the PI (LSHTM) and co-investigators.

Engagement with Policy Makers

Policy-makers both nationally and internationally will be offered the opportunity to be presented with the findings of this study. We will liaise with the National HIV programme around the viral resistance data, especially as this analysis of samples has been designed in conjunction with the National HIV program in order to meet their evidence-for-policy needs. These will include the STOP TB Partnership and the technical working groups of National TB programme and Department of HIV/AIDS of Malawi Ministry of Health. Feedback on results will also be given to Zomba hospital staff and the Department of Medicine and the district TB officers in Zomba district.

Approved by
College of Medicine
10-Dec-2020
(COMREC)
Research and Ethics Committee

16. REFERENCES

1. Ford, N. *et al.* Causes of hospital admission among people living with HIV worldwide: a systematic review and meta-analysis. *Lancet HIV* **2**, e438-444 (2015).

2. Gupta-Wright, A. *et al.* Rapid urine-based screening for tuberculosis in HIV-positive patients admitted to hospital in Africa (STAMP): a pragmatic, multicentre, parallel-group, double-blind, randomised controlled trial. *The Lancet* **392**, 292–301 (2018).

3. Gupta, R. K., Lucas, S. B., Fielding, K. L. & Lawn, S. D. Prevalence of tuberculosis in post-mortem studies of HIV-infected adults and children in resource-limited settings: a systematic review and meta-analysis. *AIDS* **29**, 1987–2002 (2015).

4. Lawn, S. D. *et al.* Diagnostic accuracy, incremental yield and prognostic value of Determine TB-LAM for routine diagnostic testing for tuberculosis in HIV-infected patients requiring acute hospital admission in South Africa: a prospective cohort. *BMC Med* **15**, 67 (2017).

5. Ford, N. *et al.* TB as a cause of hospitalization and in-hospital mortality among people living with HIV worldwide: a systematic review and meta-analysis. *J Int AIDS Soc* **19**, (2016).

6. WHO | Using the Xpert MTB/RIF assay to detect pulmonary and extrapulmonary tuberculosis and rifampicin resistance in adults and children. *WHO* https://www.who.int/tb/publications/xpert-mtb-rif-assay-diagnosis-meeting-report/en/.

7. Di Tanna, G. L. *et al.* Effect of Xpert MTB/RIF on clinical outcomes in routine care settings: individual patient data meta-analysis. *Lancet Glob Health* **7**, e191–e199 (2019).

8. Broger, T. *et al.* Novel lipoarabinomannan point-of-care tuberculosis test for people with HIV: a diagnostic accuracy study. *The Lancet Infectious Diseases* **19**, 852–861 (2019).

9. WHO | Chest radiography in tuberculosis detection. *WHO* http://www.who.int/tb/publications/chest-radiography/en/.

10. WHO | Consolidated guidelines on the use of antiretroviral drugs for treating and preventing HIV infection. *WHO* http://www.who.int/hiv/pub/arv/arv-2016/en/.

11. Qin, Z. Z. *et al.* Using artificial intelligence to read chest radiographs for tuberculosis detection: A multi-site evaluation of the diagnostic accuracy of three deep learning systems. *Sci Rep* **9**, 1–10 (2019).

12. Pande, T., Cohen, C., Pai, M. & Ahmad Khan, F. Computer-aided detection of pulmonary tuberculosis on digital chest radiographs: a systematic review. *Int. J. Tuberc. Lung Dis.* **20**, 1226–1230 (2016).

13. Harris, M. *et al.* A systematic review of the diagnostic accuracy of artificial intelligence-based computer programs to analyze chest x-rays for pulmonary tuberculosis. *PLoS One* **14**, (2019).

14. Breuninger, M. *et al.* Diagnostic accuracy of computer-aided detection of pulmonary tuberculosis in chest radiographs: a validation study from sub-Saharan Africa. *PLoS ONE* **9**, e106381 (2014).

15. Muyoyeta, M. *et al.* The sensitivity and specificity of using a computer aided diagnosis program for automatically scoring chest X-rays of presumptive TB patients compared with Xpert MTB/RIF in Lusaka Zambia. *PLoS ONE* **9**, e93757 (2014).

16. Melendez, J. *et al.* Accuracy of an automated system for tuberculosis detection on chest radiographs in high-risk screening. *Int. J. Tuberc. Lung Dis.* **22**, 567–571 (2018).

17. Rahman, M. T. *et al.* An evaluation of automated chest radiography reading software for tuberculosis screening among public- and private-sector patients. *Eur. Respir. J.* **49**, (2017).

18. Zaidi, S. M. A. *et al.* Evaluation of the diagnostic accuracy of Computer-Aided Detection of tuberculosis on Chest radiography among private sector patients in Pakistan. *Sci Rep* **8**, 1–9 (2018).

19. Philipsen, R. H. H. M. *et al.* Automated chest-radiography as a triage for Xpert testing in resource-constrained settings: a prospective study of diagnostic accuracy and costs. *Sci Rep* **5**, 12215 (2015).

Approved by College of Medicine
10-Dec-2020
(COMREC)
Research and Ethics Committee

20. MacPherson, P. *et al.* Design and protocol for a pragmatic randomised study to optimise screening, prevention and care for tuberculosis and HIV in Malawi (PROSPECT Study). *Wellcome Open Res* **3**, 61 (2018).

21. Ousley, J. *et al.* High Proportions of Patients With Advanced HIV Are Antiretroviral Therapy Experienced: Hospitalization Outcomes From 2 Sub-Saharan African Sites. *Clin Infect Dis* **66**, S126–S131 (2018).

22. Meintjes, G. *et al.* HIV-Related Medical Admissions to a South African District Hospital Remain Frequent Despite Effective Antiretroviral Therapy Scale-Up. *Medicine (Baltimore)* **94**, (2015).

23. WHO | Guidelines for managing advanced HIV disease and rapid initiation of antiretroviral therapy. *WHO* http://www.who.int/hiv/pub/guidelines/advanced-HIV-disease/en/.

24. Hakim, J. *et al.* Enhanced Prophylaxis plus Antiretroviral Therapy for Advanced HIV Infection in Africa. *New England Journal of Medicine* **377**, 233–245 (2017).

25. HIV VIROLOGIC FAILURE AND DRUG RESISTANCE AMONG HOSPITAL INPATIENTS IN MALAWI. *CROI Conference* https://www.croiconference.org/abstract/hiv-virologic-failure-and-drug-resistance-among-hospital-inpatients-in-malawi/.

26. PROSPECTIVE ENHANCED MONITORING OF DOLUTEGRAVIR-BASED FIRST LINE IN MALAWI. *CROI Conference* https://www.croiconference.org/abstract/prospective-enhanced-monitoring-of-dolutegravir-based-first-line-in-malawi/.

27. Hamada, Y., Lujan, J., Schenkel, K., Ford, N. & Getahun, H. Sensitivity and specificity of WHO's recommended four-symptom screening rule for tuberculosis in people living with HIV: a systematic review and meta-analysis. *Lancet HIV* **5**, e515–e523 (2018).

28. Chesney, M. A. *et al.* Self-reported adherence to antiretroviral medications among participants in HIV clinical trials: the AACTG adherence instruments. Patient Care Committee & Adherence Working Group of the Outcomes Committee of the Adult AIDS Clinical Trials Group (AACTG). *AIDS Care* **12**, 255–266 (2000).

29. IRIS Case Definitions | AIDS Clinical Trials Group. https://actgnetwork.org/IRIS_Case_Definitions.

30. Choko, A. T. *et al.* A pilot trial of the peer-based distribution of HIV self-test kits among fishermen in Bulisa, Uganda. *PLoS ONE* **13**, e0208191 (2018).

31. Gordon, S. B. *et al.* A Malawi guideline for research study participant remuneration. *Wellcome Open Res* **3**, 141 (2018).

32. Singer, M. *et al.* The Third International Consensus Definitions for Sepsis and Septic Shock (Sepsis-3). *JAMA* **315**, 801–810 (2016).

33. Eberhard, O. K. *et al.* Usefulness of procalcitonin for differentiation between activity of systemic autoimmune disease (systemic lupus erythematosus/systemic antineutrophil cytoplasmic antibody-associated vasculitis) and invasive bacterial infection. *Arthritis Rheum.* **40**, 1250–1256 (1997).

34. Munyati, S. S. *et al.* Chronic cough in primary health care attendees, Harare, Zimbabwe: diagnosis and impact of HIV infection. *Clin. Infect. Dis.* **40**, 1818–1827 (2005).

35. Mendelson, F. *et al.* C-reactive protein and procalcitonin to discriminate between tuberculosis, Pneumocystis jirovecii pneumonia, and bacterial pneumonia in HIV-infected inpatients meeting WHO criteria for seriously ill: a prospective cohort study. *BMC Infect. Dis.* **18**, 399 (2018).

Approved by
College of Medicine
10-Dec-2020
(COMREC)
Research and Ethics Committee

Computer Assisted Screening for Tuberculosis in Low-resource Environments (the CASTLE study)
**Principal Investigator:** Dr Rachael Burke, Malawi-Liverpool-Wellcome Trust Clinical Research Programme. PO Box 30096, Chichiri, Blantyre 3, Malawi. Tel +265 1 812 423 www.mlw.medcol.mw
**Chairperson of COMREC:** COMREC Chair, The College of Medicine of Malawi, PO Box 360, Chichiri, Blantyre 3, Malawi. Tel +265 01 874 377. www.medcol.mw

INFORMATION FORM (ENGLISH): VERSION 4.0 2020-07-30, trial arms group.

Hello, my name is ________________________. I am a Research Assistant working with the Malawi-Liverpool-Welcome Trust Clinical Research Programme in Blantyre. I am inviting you to think about taking part in a research study. This study is looking at using an x-ray picture of a person's chest, with a computer checking the x-ray picture and a urine tuberculosis test, in order to improve the diagnosis of tuberculosis.

You don't have to take part in the study; it is your choice to volunteer. If you decide not to be in this study, then you will still get all the treatment that you should get from this hospital.

Why are we doing this study?
Tuberculosis and HIV are big problems for Malawi. There are very good treatments available for both tuberculosis and HIV, but tuberculosis can be difficult to diagnose in people living with HIV. We are doing a research study in people who are living with HIV and who are admitted to hospital, in order to see if taking an x-ray picture of a person's chest, and having that x-ray checked by a computer, as well as a urine test can help us diagnose and treat tuberculosis more quickly and improve people's health. We also want to find out about how ART drugs are working in Malawi.

Why are we asking you to take part in the study?
You have been asked to take part because you need admission to hospital and are living with HIV.

What will happen to me if I agree to take part in the study?
If you agree to take part in the study, we will ask you to cough into a small pot so that we can check for tuberculosis. The research team will also look at your medical records while you are in hospital to find out whether the hospital medical team started you on tuberculosis treatment or not.

Half of the people who agree to be in this study will be asked to have an x-ray picture of their chest and provide a urine sample. The x-ray picture does not hurt at all, and will take a short time to complete.

If you have a chest x-ray, a computer programme will check the chest x-ray to see whether there might be tuberculosis in your lungs. If the x-ray suggests that there might be TB we will ask you again to cough into a small cup and we will test your sputum for tuberculosis. If you are found to have tuberculosis, your healthcare team in the hospital will start you on tuberculosis treatment.

Half the people in the study will not have the chest x-ray. Whether of not you have a chest x-ray depends on the day you are admitted to hospital. If you agree to take part in the study you will not be able to choose whether you have a chest x-ray or not.

If you wish, you can optionally, provide a blood test sample to see if HIV is detected in your blood ("HIV viral load"). If you don't want to provide a blood sample, you can still be in the Chest Xray study.

If you agree, we will take 12mL of blood (3 teaspoons) to test for HIV viral load and tests about ART. We will keep a small sample of blood for up to 5 years in order to develop new blood tests to diagnose tuberculosis. Some of your samples might be transported out of Malawi to other countries to do tests about how ART drugs work.

Approved by
College of Medicine
10-Dec-2020
(COMREC)
Research and Ethics Committee

One of the tests we will do on your sputum for tuberculosis takes several weeks before the result is ready. If this test shows you have tuberculosis and you have not already started on tuberculosis treatment we will contact you by telephone to let you know to come to the tuberculosis clinic. We will also contact you or your relative by telephone in 8 weeks time to find you how you are getting on. If we cannot reach you or a relative by telephone, we may come to visit your house to find out how you are. If you have a blood test and you have HIV virus detectable in your blood today, we will ask you to come back to the hospital in 8 weeks time for another blood test to see if the HIV virus is still detectable.

All people in this study will get treatment for HIV and tuberculosis (if they need tuberculosis treatment) in the same way as any other patient in the hospital. HIV treatment and tuberculosis treatment is available and free of charge in Malawi.

Are there any risks for me from being in this study?
There are no big risks. The chest x-ray is safe, with an extremely low risk of causing any health problems. If you choose to give a blood sample, this might cause discomfort.

Are there any benefits for me from being in this study?
There are no clear benefits to you from being in the study. We don't know yet whether taking a chest x-ray picture with computer checking and a urine TB test makes a difference to people; that's why we want to do the study. You will get any treatment you need for tuberculosis from the hospital whether or not you choose to be in the study.

Reimbursement
We will give you MKW 1000 for participating in the study.

Protecting privacy and data confidentiality
We will protect your privacy by only asking study questions where no-one else can hear.

The people working for the study will keep your identity and tuberculosis test results secret, so that only people working for the study and the hospital will see them. We will store information about you (including the picture of your chest from the x-ray) on a computer protected by a password. Staff working at Zomba hospital will be able to see your chest x-ray picture so that they can give you clinical care. Staff from the Xray service provider (Thirona and minXray) might also see your X-rays in order to provide technical support to the X-ray service. The X-ray service provider (Thirona) will receive information about your tuberculosis test results in order to make the computer better at detecting tuberculosis for people like you in the future. Staff from London School of Hygiene and Tropical Medicine and the Malawi College of Medicine might also see your information as they monitor the trial.

We will share anonymous data. Anonymous means that your name is not included in anything that we share and that the information cannot be linked back to you.

What happens if you want to leave the study early?
You are free to leave the study at any time if you choose. If you decide not to be in the study, or if you leave the study early, then you will still get all the treatment that you should get from this hospital.

Who do I call if I have questions or problems?
You can ask the research assistant who gave you this consent form, or you can call Dr Rachael Burke or Dr Marriott Nliwasa at MLW on Tel +265 1 812 423 if you have questions, complaints, or get sick or injured as a result of being in this study. You can also contact the College of Medicine Research Ethics Committee Chairman; COMREC Secretariat, College of Medicine, P/Bag 360, Chichiri, Blantyre 3, Malawi; Tel: +265 1871911 ext. 334; E-mail: comrec@medcol.mw

Can I ask questions?

Approved by
College of Medicine
10-Dec-2020
(COMREC)
Research and Ethics Committee

If the research assistant who gave you this form cannot answer your questions, then you are free to call Dr Rachael Burke or Dr Marriott Nliwasa on +265 1 812 423

What if something goes wrong?

The London School of Hygiene and Tropical Medicine holds insurance policies which apply to this study. If you experience harm or injury as a result of taking part in this study, you may be eligible to claim compensation without having to prove that the School is at fault. This does not affect your legal rights to compensation

Approved by
College of Medicine
10-Dec-2020
(COMREC)
Research and Ethics Committee

Computer Assisted Screening for Tuberculosis in Low-resource Environments (the CASTLE study)
**Principal Investigator:** Dr Rachael Burke, Malawi-Liverpool-Wellcome Trust Clinical Research Programme. PO Box 30096, Chichiri, Blantyre 3, Malawi. Tel +265 1 812 423 www.mlw.medcol.mw
**Chairperson of COMREC:** COMREC chair, The College of Medicine of Malawi, PO Box 360, Chichiri, Blantyre 3, Malawi. Tel +265 01 874 377. www.medcol.mw

Participant ID:

CONSENT FORM (ENGLISH): VERSION 4.0 2020-07-30, trial arms group.

Please tick all boxes

1. I have received and read / had the information sheet read to me by the researcher that explains in detail the reasons for the study (CASTLE PIL trial arms v4.0 2020-07-30)	
2. I understand that the study involves research.	
3. I have asked all the questions that I have about the purpose of the research study and feel happy that I have enough information about the study.	
4. I agree that, if I agree to participate, I will be allocated to either a chest x-ray with computer checking the x-ray result and a urine sample, or to usual care and I won't get to pick which one I receive.	
5. I agree that, if I agree to participate, the researchers and authorised individuals from College of Medicine and London School of Hygiene and Tropical Medicine will be able to look at my medical records.	
6. I agree that, if I agree to participate, information might be shared with X-ray service providers (Thirona and minXray).	
7. I agree that, if I agree to participate, the researchers may contact me or a family member by telephone or by home visit.	
8. I know that any information about me where I can be identified will be kept secret.	
9. If I do not agree to take part in this study, I understand that I will not be penalised for doing so by the researcher, nor by any medical service providers in the future.	
10. I understand that I may discontinue participation in the study at any time without penalty.	
11. OPTIONAL: I agree that researchers may take a blood sample for tests about HIV, ART medicines and TB.	
12. OPTIONAL: I agree that, if I agree to participate, the researchers may transport my blood sample outside of Malawi.	

Should you have any questions at any time, please contact the lead researcher: Dr Rachael Burke, or the Chair of COMREC at the address or telephone numbers above.

I voluntarily agree to take part in this study

**Name of Participant (BLOCK CAPITALS)**	**Date** (DD-MON-YYYY)	**Signature or thumb print**
**Name of Witness (BLOCK CAPITALS)** **[Required if participant unable to read / write]**	**Date** (DD-MON-YYYY)	**Signature**
**Name of Researcher (BLOCK CAPITALS)**	**Date** (DD-MON-YYYY)	**Signature**

Complete two copies: Give one to participant to keep. Place one in participant's study folder.

Approved by College of Medicine
10-Dec-2020
(COMREC)
Research and Ethics Committee

Computer Assisted Screening for Tuberculosis in Low Resource Environments (the CASTLE study)
**Principal Investigator:** Dr Rachael Burke, Malawi-Liverpool-Wellcome Trust Clinical Research Programme. PO Box 30096, Chichiri, Blantyre 3, Malawi. Tel +265 1 812 423 www.mlw.medcol.mw
**Chairperson of COMREC:** COMREC chair, The College of Medicine of Malawi, PO Box 360, Chichiri, Blantyre 3, Malawi. Tel +265 01 874 377. www.medcol.mw

INFORMATION FORM (ENGLISH): VERSION 4.0 2020-07-30, diagnostic cohort group.

Hello, my name is _________________________. I am a Research Assistant working with the Malawi-Liverpool-Welcome Trust Clinical Research Programme in Blantyre. I am inviting you to think about taking part in a research study. This study is looking at using an x-ray picture of a person's chest, with a computer checking the x-ray picture, and a urine tuberculosis test in order to improve the diagnosis of tuberculosis. The study is also to find out about the types of illness that cause people living with HIV to get sick and come to hospital.

You don't have to take part in the study; it is your choice to volunteer. If you decide not to be in this study, then you will still get all the treatment that you should get from this hospital.

Why are we doing this study?
Tuberculosis and HIV are big problems for Malawi. There are very good treatments available for both tuberculosis and HIV, but tuberculosis can be difficult to diagnose in people living with HIV. We are doing a research study in people who are living with HIV and who are admitted to hospital, in order to see if taking an x-ray picture of a person's chest, and having that x-ray checked by a computer, as well as a urine test, can help us diagnose and treat tuberculosis more quickly and improve people's health.

We also want to find about the types of illness that cause people living with HIV to get sick and come to hospital, in order that we can develop better ways of looking after people living with HIV in hospital.

Why are we asking you to take part in the study?
You have been asked to take part because you need admission to hospital and are living with HIV.

What will happen to me if I agree to take part in the study?
If you agree to take part in the study, we will ask you a series of questions about your health, which will take about 20 – 30 minutes of your time. The research team will also look at your medical records while you are in hospital to find out what sort of treatments you have.

We will ask you to cough sputum into a small pot so that we can check for tuberculosis. We will ask you to give a urine sample. We will also take 22mL of blood (4 – 5 teaspoons) to test for various different diseases and markers of disease. Most of the blood will be used to do tests for tuberculosis and other infectious diseases straight away. If you agree, we will also keep a small sample of blood, urine and sputum for up to 5 years in order to develop new blood tests to diagnose tuberculosis. Some of your samples might be transported out of Malawi to other countries to do tests about how HIV drugs work.

We will take a picture of your lungs with a chest x-ray and a computer programme will check the chest x-ray to see whether there might be tuberculosis in your lungs. If the computer programme says you are likely to have tuberculosis we will ask to cough another sample of sputum into a pot. The x-ray picture does not hurt at all and will take a very short time to complete.

If you agree, we will phone you or your relative after 56 days to see how you are getting on. If we cannot reach you or a relative by telephone, we may come to visit your house to find out how you are.

Approved by College of Medicine 10-Dec-2020 (COMREC) Research and Ethics Committee

One of the tests we will do on your sputum for tuberculosis takes several weeks before the result is ready. If this test shows you have tuberculosis and you have not already started on tuberculosis treatment we will contact you by telephone to let you know to come to the tuberculosis clinic.

All people in this study have treatment for HIV, tuberculosis (if they need tuberculosis treatment) and other infections (if they need it) in the same way as any other patient in the hospital. HIV treatment, tuberculosis treatment and treatment for other infections is available and free of charge in Malawi.

Are there any risks for me from being in this study?
There are no big risks. The chest x-ray is safe, with an extremely low risk of causing any health problems. The blood test might cause discomfort.

Are there any benefits for me from being in this study?
There are no clear benefits to you from being in the study. We don't know yet whether taking a chest x-ray picture with computer checking and a urine test makes a difference to people; that's why we want to do the study.
You will get any treatment you need for HIV, tuberculosis and other infections from the hospital whether or not you choose to be in the study.

Reimbursement
We will give you MKW 8000 for participating in the study.

Protecting privacy and data confidentiality
We will protect your privacy by only asking study questions where no-one else can hear.

The people working for the study will keep your identity and tuberculosis test results secret, so that only people working for the study and the hospital will see them. We will store information about you (including the picture of your chest from the x-ray) on a computer protected by a password. Staff working at Zomba hospital will be able to see your chest x-ray picture so that they can give you clinical care. Staff from the Xray service provider (Thirona and minXray) might also see your X-rays in order to provide technical support to the X-ray service. The X-ray service provider (Thirona) will receive information about your tuberculosis test results in order to make the computer better at detecting tuberculosis for people like you in the future. Staff from London School of Hygiene and Tropical Medicine and the Malawi College of Medicine might also see your information as they monitor the trial.

We will share anonymous data. Anonymous means that your name is not included in anything that we share and that the information cannot be linked back to you.

What happens if you want to leave the study early?
You are free to leave the study at any time if you choose. If you decide not to be in the study, or if you leave the study early, then you will still get all the treatment that you should get from this hospital.

Who do I call if I have questions or problems?
You can ask the research assistant who gave you this consent form, or you can call Dr Rachael Burke or Dr Marriott Nliwasa at MLW on +265 1 812 423 if you have questions, complaints, or get sick or injured as a result of being in this study. You can also contact COMREC Secretariat, College of Medicine, P/Bag 360, Chichiri, Blantyre 3, Malawi; Tel: +265 1871911 ext. 334; E-mail: comrec@medcol.mw.

Can I ask questions?
You are free to ask any questions you have at any time. If the research assistant who gave you this form cannot answer your questions, then you are free to call Dr Rachael Burke or Dr Marriott Nliwasa on +265 1 812 423

Approved by College of Medicine
10-Dec-2020
(COMREC)
Research and Ethics Committee

What if something goes wrong?

The London School of Hygiene and Tropical Medicine holds insurance policies which apply to this study. If you experience harm or injury as a result of taking part in this study, you may be eligible to claim compensation without having to prove that the School is at fault. This does not affect your legal rights to compensation.

Approved by
College of Medicine
10-Dec-2020
(COMREC)
Research and Ethics Committee

Computer Assisted Screening for Tuberculosis in Low-resource Environments (the CASTLE study)
**Principal Investigator:** Dr Rachael Burke, Malawi-Liverpool-Wellcome Trust Clinical Research Programme. PO Box 30096, Chichiri, Blantyre 3, Malawi. Tel +265 1 812 423 www.mlw.medcol.mw
**Chairperson of COMREC:** COMREC chair, The College of Medicine of Malawi, PO Box 360, Chichiri, Blantyre 3, Malawi. Tel +265 01 874 377. www.medcol.mw

Participant ID:

CONSENT FORM (ENGLISH): VERSION 4.0 2020-07-30, diagnostic cohort group.

Please tick all boxes

1. I have received and read / had the information sheet read to me by the researcher that explains in detail the reasons for the study (CASTLE PIL cohort v4.0 2020-07-30)	
2. I understand that the study involves research.	
3. I have asked all the questions that I have about the purpose of the research study and feel happy that I have enough information about the study.	
4. I agree that, if I agree to participate, the researchers and authorised individuals from College of Medicine and London School of Hygiene and Tropical Medicine will be able to look at my medical records.	
5. I agree that, if I agree to participate, information might be shared with X-ray service providers (Thirona and minXray).	
6. I agree that, if I agree to participate, the researchers may store blood samples for later research about how to diagnose tuberculosis.	
7. I agree that, if I agree to participate, the researchers may transport my samples outside of Malawi.	
8. I agree that, if I agree to participate, the researchers may contact me or a family member of my choosing by telephone or by home visit.	
9. I know that my information where I can be identified will be kept secret	
10. If I do not agree to take part in this study, I understand that I will not be penalised for doing so by the researcher, nor by any medical service providers in the future.	
11. I understand that I may discontinue participation in the study at any time without penalty.	

Should you have any questions at any time, please contact the lead researcher (Dr Rachael Burke) or the Chair of COMREC at the address or telephone numbers above.

I voluntarily agree to take part in this study

Name of Participant (BLOCK CAPITALS) | **Date (**DD-MON-YYYY) | **Signature or thumb print**

Name of Witness (BLOCK CAPITALS) | **Date (**DD-MON-YYYY) | **Signature**
[required if participant unable to read / write]

Name of Researcher (BLOCK CAPITALS) | **Date (**DD-MON-YYYY) | **Signature**

Complete two copies: Give one to participant to keep. Place one in participant's study folder.

Approved by College of Medicine
10-Dec-2020
(COMREC) Research and Ethics Committee

Computer Assisted Screening for Tuberculosis in Low-resource Environments (the CASTLE study)
**Mkulu wakafukufuku:** Dr Rachael Burke, Malawi-Liverpool-Wellcome Trust Clinical Research Programme. PO Box 30096, Chichiri, Blantyre 3, Malawi. Tel Tel +265 1 812 423 www.mlw.medcol.mw
**Mkulu wa bungwe loyang'anira kafukufuku la COMREC** : COMREC chair, The College of Medicine of Malawi, PO Box 360, Chichiri, Blantyre 3, Malawi. Tel +265 1 871 911. www.medcol.mw

KALATA YA UTHENGA OKHUDZA KAFUKUFUKU (CHICHEWA): VERSION 4.0 2020-07-30, trial arms group.

Mulibwanji? Dzina langa ndine_______________. Ndine ogwira ntchito mukafukufuku ochokera ku bungwe la Malawi-Liverpool-Welcome Trust Clinical Research Programme mu Blantyre. Ndikukupemphani kuti muganizire ngati mufuna kutenga nawo mbali mu kafukufukuyu. Cholinga chakafufukuyi ndichoti tione pogwiritsa nticchito chithunzi cha x-ray chachifuwa cha munthu ndi makina a kompyuta, kuyanang'ana chimene chitunzi cha x-ray chawonetsa, komanso kuyeza matenda a TB m'mikozo; kuti tipitise patsogolo zofufuzira matenda a chifuwa chachikulu cha TB.

Sizokakamiza kuti inu mutenge nawo mbali mukafukufukuyu, ndichisankho chanu kulora kutero. Ngati simulora kutenga nawo mbali mukafukufukuyu, mudzalandirabe chithandizo chonse chomwe mumayenera kulandira kuchipatala kuno.

Ndi chifukwa chiyani tikupanga kafukufukuyu?

Matenda a chifuwa chachikulu ndi kachilombo ka HIV amabweretsa mavuto ambiri azaumoyo kuno ku Malawi. Pali njira zosiyanasiyana zabwino zachithandizo cha mankhwala a chifuwa chachikulu ndi kachilombo ka HIV, koma chifuwa chachikulu chimavuta kuchipeza pakati pa anthu omwe ali ndi kachirombo cha HIV. Tikuchita kafukufuku pakati pa anthu omwe ali ndi HIV komanso anawagonekapo m'chipatala. Tikuchita izi kuti tiwone ngati kutenga chithunzi cha x-ray cha chifuwa cha munthu ndi kugwiritsa ntchito kompyuta oyang'anira zithunzizo, komanso kugwiritsa ntchito mikozo poyeza matenda a TB, kungatithandize kuzindikira ndi kuchiza chifuwa chachikulu mofulumira komanso kusintha thanzi la anthu. Kafukufukuyu athandizanso kuti tidziwe mmene mankhwala a ma ARV akuyendela m'Malawi muno.

Nchifukwa chiyani tikukufunsani kuti mutenge nao mbali mu kafukufukuyu?
Takufusani kuti mutenge nao mbali mu kafukufukuyu chifukwa mukuyenera kugona mu chipatala komaso muli ndi kachirombo ka HIV.

Kodi chingachitike ndi chiyani ngati ndavomereza kutenga nawo mbali mu kafukufukuyu?
Ngati mwalolera kutenga nawo mbali mukafukufukuyu, tizakupemphani kuti mukosomore ndi kuthila makholol0 anu mukabotolo kuti tifufuze matenda a chifuwa chachikulu. Gulu logwira ntchito mukafukufukuyu liwonanso mabuku anu zachipatala kuti adziwe ngati anthu ogwira ntchito muchipatala anakuyambitsani mankhwala okhudza chifuwa chachikulu kapena ayi.

Theka la anthu omwe avomereza kukhala mu kafukufukuyu adzafunsidwa kuti akhale ndi chithunzi cha x-ray cha m'chifuwa chawo komanso kuti apeleke mkozo kuti uyezedwe. Kujambulidwa ndi x-ray sikumapweteka nkomwe, ndipo chidzatenga nthawi yaying'ono kuti zonse zitheke. Makina a X-ray ali m'chipinda choyandikira.

Ngati mujambulidwa ndi x-ray, pulogalamu ya pakompyuta idzafufuza chithunziyo ngati pangakhale chifuwa chachikulu m'mapapo anu. Ngati x-ray ikusonyeza kuti pakhoza kukhala TB tikufunsaninso kuti mukhosomoleso mu kapu yaing'ono kuti tiyeze makhololo anu ngati muli kachilombo ka TB.

Approved by
College of Medicine
10-Dec-2020
(COMREC)
Research and Ethics Committee

Ngati mwapezeka ndi chifuwa chachikulu, akuchipatala adzakuyambitsani kulandila mankwala ochiritsa chifuwa cha chikulu.

Theka la anthu mu kafukufukuyu sadzajambulidwa ndi x-ray. Kaya mwajambulidwa ndi x-ray kapena ayi, zitengela tsiku limene anakugonekani ku chipatala. Ngati mutavomere kutenga mbali mukafukufukuyi simutha kusankha ngati mujambulidwa ndi x-ray kapena ayi.

Tizakhalanso tikuyeza mulingo wa kachilomba ka HIV mthupi. Muzakhala ndi chisankho cho peleka nawo magazi kuti zimenezi ziyezedwe. Ngati simuzafuna, muzakhalabe ndi mwayi otenga nawo mbali mukafukufuku wa chithunzi cha x-ray.

Ngati muzasankhe kuyedzedwa nawo mulingo wa ka chilombo ka HIV mthupi, tizatenga magazi okwana 12mL (ma supuni an'gono atatu) kuti tiyeze zimenezi komanso zoyeza zokhuzana ndi mankhwala a ma ARV. Magaziwa azasungindwa kwa zaka zokwana zisanu (5) kuti zitithandize kupanga njila zina zoyezela matenda a TB. Magazi ena azatumizidwa ku mayiko akunja kuti akayeze zokhuzana ndi mankhwala a ma ARV pa makina amakono.

Chimodzi mwa zoyeza chifuwa cha chikulu mu makhololo anu chimatenga masabta angapo kuti zotsatira zituluke. Ngati zotsatira zanu zawonetsa kuti muli ndi chifuwa chachikulu komanso simunayambe kulandira mankwala tidzakuyimbirani foni kuti mubwere kuchipatala kuti mulandire chithandizo cha chifuwa chachikulu. Tizayimbanso foni kwa inu kapena abale anu pakatha ma sabata asanu ndi zitatu (8) kuti timve mmene mukuchitira. Ngati tingalephere kukupezani palamya yanu yammanja, tizatha kukuyenderani kwanu kuti timve mmene mukuchitira. Ngati takuyezani mulingo wa ka chilombo ka HIV mthupi nkupeza kuti ndikokwela, tizakuwuzani kuti mubwelenso kuchipatala pakatha ma sabata asanu ndi zitatu (8) kuti tizayezenso ngati ka chilomboko kakupezekabe.

Anthu onse mu kafukufukuyu adzalandira chithandizo cha HIV ndi chifuwa chachikulu (ngati ayenera kulandira chithandizo cha chifuwa chachikulu) mofanana ndi wina aliyense m'chipatala. Thandizo la HIV ndi chifuwa cha chikulu likupezeka kwaulere ku Malawi.

Kodi pali chiopsezo china chilichonse potenga nawo mbali mukafukufukuyu?
Palibe chiopsezo chachikulu chinachilichonse. Njira yojambula mu chifuwa ya x-ray ndi yotetezeka ndipo chiwopsezo chokuti njirayi itha kuyambitsa mavuto pa umoyo wanu ndichochepa kwambiri. Kupeleka magazi kumatha kupweteka.

Kodi pali zopindula zinazilizonse potenga nawo mbali mukafukufukuyu?
Palibe chopindula chinachilichonse choonekeratu chimene inu mulandire potenga nawo mbali mukafukufukuyu. Sitikudziwabe ngati kugwiritsa ntchito chitunzi cha x-ray cha chifuwa limodzi ndi kompyuta oyang'anira zithunziso komanso kuyeza TB m'mikozo kutha kupititsa patsogolo umoyo wa anthu; ndi chifukwa chake tikufuna kuchita kafukufukuyu. Mudzalandilabe chitandizo chokhudzana ndi chifuwa chachikulu yoyenera ngati wasanka kapena simunasankhe kutenga nawo mbali mu kafukufukuyu.

Zokhudza chipuputa misonzi
Tizakupatsani ndalama zokwana MWK1000 potenganawo mbali mukafufukuyu.

Zokhudza chinsinsi ndi chitetezo cha mauthenga anu
Tidzateteza mauthenga anu poonetsetsa kuti tikukufunsani mafunso akafukufuku pamalo okuti palibe wina amene angamvetsere zokambirana zathu.

Anthu omwe akugwira ntchito mukafukufuku adzasunga chizindikiro ndi zotsatira zanu mwa chinsisi, kuti ndi anthu akafukufuku ndi akuchipatala amene angathe kuona mauthengawo. Tidzasunga mauthenga onse

Approved by College of Medicine
10-Dec-2020
(COMREC) Research and Ethics Committee

akafukufuku (kuphatikizapo chithunzi cha mchifuwa mwanu kuzera makina a x-ray) pa kompyuta yotetezedwa ndi mawu achinsinsi. Anthu ogwira nchito kuchipatala cha Zomba azakhala ndi mwayi owona chithunzi cha mchifuwa mwanu cha X-ray kuti azathe kukupasani chithandizo chamankhwala moyenerera. Ogwira ntchito kumakampani a makina a x-ray (ku Thirona ndi MinXray) azathanso kuwona zithunzizo kuti azitha kutithandiza powonesesa kuti makina a x-ray akugwila ntchito bwinolomwe. Ogwira ntchito kukampani ya makina a X-ray ya Thirona azathanso kuwona zosatila zanu za chifuwa cha chikulu cha TB kuti ziwathandize kuwonjezera mphamvu ya kopyuta kuwunika matenda a TB mwa anthu. Akadaulo a ku ma sukulu a ukachendede a London School of Hygiene and Tropical Medicine ndi College of Medicine yaku Malawi azathanso kuwona uthenga wokhuzana ndi inu, kuti ziwathandize kuwonesesa kuti kafukufukuyu akuyenda bwino.

Mauthenga amene azagawidwe adzakhala opanda chizindikiro chanu. Ndikutanthauza kuti dzina lanu silidzaphatikizidwa mu chilichonse timagawana komanso palibe amene angadziwe kuti ndi inuyo amene mwatipasa mauthengawo.

Nanga chingachitike ndi chiyani ngati mukufuna mutuluke mukafukufuku nthawi isanakwane?
Muli ndi ufulu kutuluka mukafukufukuyu pa nthawi inailiyonse yomwe inu mungafune. Ngati musakufuna kutenga nawo mbali mukafukufukuyu, kapena mwatuluka mukafukufukuyu mwamsanga nthawi yake isanakwane, ndiye kuti inu mudzathabe kulandira chithandizo china chili chonse choyenera pachipatala pano.

Tigaimbire foni ndani patakhala mafunso kapena zovuta zina?
Mutha kufunsa munthu wakafukufuku amene wakupatsani chikalatachi kapena mutha kuimbira foni a Dr Rachael Burke kapena Dr Marriott Nliwasa aku MLW pa nambala iyi +265 1 812 423 ngati muli ndi mafunso, zodandaula, kapena mwadwala kapena mwapweteka chifukwa chotenga nawo mbali mukafukufukuyu. Muthanso kuimbira mkulu wa bungwe COMREC Secretariat, College of Medicine, P/Bag 360, Chichiri, Blantyre 3, Malawi; Nambala ya lamya: +265 1871911 ext. 334; Kotumizira ma uthenga a imelo: comrec@medcol.mw

Kodi nditha kufunsa mafunso?
Muli omasuka kufunsa mafunso amene muli nawo nthawi inailiyonse. Ngati munthu wakafukufuku amene anakupatsani uthengawu sangathe kukuyankhani mafunso anu, ndiye kuti muli ololedwa kuwaimbira foni a Dr Rachael Burke kapena Dr Marriott Nliwasa pa +265 1 812 423

Chingachitidwe chiyani ngati chinachake chalakwika?

Sukulu yaukadaulo (University), The London School of Hygiene and Tropical Medicine, amakhala ndi inshuwalansi yomwe amapezeka pa kafukufukuyu. Ngati mukapwetekedwa kapena mukavulazidwa chifukwa cha kutenga nawo mbali mu kafukufukuyu, mukhoza kulandira chopepesa popanda kuwonetsa kuti sukulu yaukadulo ndiwolakwitsa. Izi sizikhudza ufulu wanu walamulo kuti mulandira chopepets

Approved by
College of Medicine
10-Dec-2020
(COMREC)
Research and Ethics Committee

Participant ID:

**Computer Assisted Screening for Tuberculosis in Low-resource Environments (the CASTLE study)**
**Mkulu wakafukufuku:** Dr Rachael Burke, Malawi-Liverpool-Wellcome Trust Clinical Research Programme. PO Box 30096, Chichiri, Blantyre 3, Malawi. Tel +265 1 812 423 www.mlw.medcol.mw
**Mkulu wa bungwe loyang'anira kafukufuku la COMREC**: Dr YB Mlombe, The College of Medicine of Malawi, PO Box 360, Chichiri, Blantyre 3, Malawi. Tel +265 1 871 911. www.medcol.mw

KALATA YA CHILOLEZO CHOTENGA NAWO MBALI MUKAFUKUFUKU: VERSION 4.0 2020-07-30, trial arms group

Chongani mabokosi onse

1.	Ndalandira ndipo ndawelenga zokhudza kafukufuku/ ndipo opanga kafukufukuyu afotokoza momveka bwino zolinga zakafukufukuyu (CASTLE PIL trial arms v4.0 2020-07-30)	
2.	Ndamvetsetsa kuti zochitika izi ndi zakafukufuku.	
3.	Ndafunsa mafunso onse amene ndinali nawo okhudza cholinga chakafukufukuyu ndipo ndili okhutira kuti ndili ndi uthenga onse ofunika okhudza kafukufukuyu.	
4.	Ndikuvomera kuti ndikapereka chilolezo chotenga nawo mbali mukafukufukuyu akandiyika mugulu limene lidzajambulidwa pa chifuwa ndi x-ray yophatikiza kompyuta yokayang'ana zotsatirazo komanso kupeleka mikozo, kapena akandiyika mugulu lomwe sindidzajambulidwa koma ndidzalandira chithandizo chachizolowezi. Ndipo ine sindidzakhala ndisankho ya njira imene ndidzalandire.	
5.	Ndikuvomereza kuti, ngati ndavomera kutenga nawo mbali mu kafukufukuyu, akafukufuku ndi akadaulo ena ochokera kusukulu yaokachenjede ya College of Medicine ndi London School of Hygiene and Tropical Medicine adzayang'ana ma rekodi anga azachipatala.	
6.	Ndikuvomereza kuti, ngati ndavomera kutenga nawo mbali mu kafukufukuyu, ma uthenga okhudzana ndi ine atha kuzagawidwa kwa akumakampani a x-ray (a Thirona ndi MinXray)	
7.	Ndikuvomereza kuti, ngati ndavomera kutenga nawo mbali mu kafukufukuyu, akafukufuku atha kundiimbira inuyo kapena achibale anu foni kuti tilankhulane kapena kukuyenderani kwanu.	
8.	Ndikudziwa kuti mauthenga onse okhudzana ndi ine adzasungidwa mwachinsinsi ndi mosadziwika kuti uthengawu wachokera kwa ine.	
9.	Ngati sindingavomereze kutenga nawo mbali mu kafukufukuyu, ndamvetsetsa kuti sindidzaweruzidwa ndi akafukufuku kapena akuchipatala m'tsogolomu.	
10.	Ndamvetsetsa kuti ndithakusiya kutenga nawo mbali mukafukufukuyu nthawi inailiyonse popanda chilango.	
11.	MWAKUFUNA KWANU: Ndavomela kuti akafukufuku anditenge magazi kuti akayeze zokhuzana ndi kachilombo ka HIV, mankhwala a ma ARV, ndi matenda a TB	
12.	MWAKUFUNA KWANU: Ndavomela kuti kuvomela kwanga kuloleza kuti akafukufuku atumize magazi anga kumayiko akunja kwa dziko la Malawi.	

Ngati muli ndi funso linalilionse nthawi ina, chonde yankhulani ndi mkulu wa kafukufukuyu: Dr Rachael Burke, kapena mkulu wa bungwe COMREC pa adiresi ndi nambala za foni zilipamwambazo.

PTO

Approved by
College of Medicine
10-Dec-2020
(COMREC)
Research and Ethics Committee

Participant ID:

Ndalolera mwakufuna kwanga kutenga nawo mbali mukafukufukuyu

**Dzina lanu, (MUZILEMBO ZAZIKULU)**	**Tsiku (DD-MON-YYYY)**	**Sayini kapena chidindo cha chala**
**Dzina la mboni (MUZILEMBO ZAZIKULU)**<br>**(Ngati satha kulemba/ kuwerenga)**	**Tsiku (DD-MON-YYYY)**	**Sayini**
**Dzina la wakafukufuku(MUZILEMBO ZAZIKULU)**	**Tsiku (DD-MON-YYYY)**	**Sayini**

Sayinani pa mapepala awili: pepala limodzi mupatse otenga nawo mbali mukafukufuku. Ina yokuti isungidwe limodzi ndimapepala ena akafukufuku

Approved by
College of Medicine
10-Dec-2020
(COMREC)
Research and Ethics Committee

Computer Assisted Screening for Tuberculosis in Low Resource Environments (the CASTLE study)
**Mkulu wakafukufuku:** Dr Rachael Burke, Malawi-Liverpool-Wellcome Trust Clinical Research Programme. PO Box 30096, Chichiri, Blantyre 3, Malawi. Tel +265 1 812 423 www.mlw.medcol.mw
**Mkulu wa bungwe loyang'anira kafukufuku la COMREC** : Dr YB Mlombe, The College of Medicine of Malawi, PO Box 360, Chichiri, Blantyre 3, Malawi. Tel +265 1 871 911. www.medcol.mw

KALATA YA UTHENGA OKHUDZA KAFUKUFUKU (CHICHEWA): VERSION 4.0 2020-07-30, diagnostic cohort group

Mulibwanji? Dzina langa ndine________________. Ndine ogwira ntchito mukafukufuku ochokera ku bungwe la Malawi-Liverpool-Welcome Trust Clinical Research Programme mu Blantyre. Ndikukupemphani kuti muganizire ngati mufuna kutenga nawo mbali mu kafukufukuyu. Cholinga chakafufukuyi ndichoti tione pogwiritsa nticchito chithunzi cha x-ray pa chifuwa cha munthu ndi kompyuta kuyanang'ana chimene chithunzi cha x-ray chawonetsa komanso kuyeza matenda a TB m'mikozo; kuti tipitise patsogolo zofufuzira matenda a chifuwa chachikulu cha TB.. Kafukufukuyu tikufunanso kudziwa za mitundu ya matenda omwe amachititsa kuti anthu omwe ali ndi kachilombo ka HIV adwale ndikubwera ku chipatala.

Sizokakamiza kuti inu mutenge nawo mbali mukafukufukuyu, ndichisankho chanu kulora kutero. Ngati simulora kutenga nawo mbali mukafukufukuyu, mudzalandirabe chithandizo chonse chomwe mumayenera kulandira kuchipatala kuno.

Ndi chifukwa chiyani tikupanga kafukufukuyu?

Matenda a chifuwa chachikulu ndi kachilombo ka HIV amabweretsa mavuto ambiri azaumoyo kuno ku Malawi. Pali njira zosiyanasiyana zabwino zachithandizo cha mankhwala a chifuwa chachikulu ndi kachilombo ka HIV, koma chifuwa chachikulu chimavuta kuchipeza pakati pa anthu omwe ali ndi kachirombo cha HIV. Tikuchita kafukufuku pakati pa anthu omwe ali ndi HIV komanso anawagonekapo m'chipatala. Tikuchita izi kuti tiwone ngati kutenga chithunzi cha x-ray cha chifuwa cha munthu ndi kugwiritsa ntchito kompyuta oyang'anira zithunzizo, kungatithandize kuzindikira ndi kuchiza chifuwa chachikulu mofulumira komanso kusintha thanzi la anthu.

Tikufunanso kufufuza za matenda omwe amachititsa anthu amwe ali ndi kachirombo ka HIV kuti adwale ndikubwera kuchipatala, kuti titha kupeza njira zabwino zosamala anthu omwe ali ndi HIV kuchipatala

Nchifukwa chiyani tikukufunsani kuti mutenge nao mbali mu kafukufukuyu?

Takufusani kuti mutenge nao mbali mu kafukufukuyu chifukwa mukuyenera kugona mu chipatala komanso muli ndi kachirombo ka HIV.

Kodi chingachitike ndi chiyani ngati ndavomereza kutenga nawo mbali mu kafukufukuyu?

Ngati mungalole kutenga nawo mbali mukafukufukuyu, tiikufunsani mafunso angapo okhudzana ndi umoyo wanu. Mafunsowa atenga mphindi zokwana 20-30. Gulu logwira ntchito mukafukufukuyu liwonanso mabuku anu achipatala kuti adziwe mankhwala amene mukulandira.

Tikupemphani kuti mukhosomole ndi kuthira makhololo anu mu kakapo kakan'gono komanso kupeleka mkozo kuti tifufuze matenda a chifuwa chachikulu cha TB. Tidzatengaso magazi okwanira 22ml (masipuni ang'ono okwana 4 kapena 5) kuti tiyezere matenda osiyanasiyana ndi zizindikiro za mathenda. Magazi ambiri adzagwiritsidwa ntchito poyezera chifuwa chachikulu ndi matenda ena opatisirana. Ngati mwavomera kutenga nawo mbali mu kafukufukuyu, tidzasunga Magaziwo, mikozo ndi makhololo kwazaka zisanu kuti tipeze mayezedwe atsopano a magazi ofufuzira chifuwa chachikulu. Komanso pazatha kokhala kufunikira kutumiza zoyezazo kunja kuti zikayezedwe ndi makina ena omwe kuno tilibe.

Approved By
College of Medicine
10-Dec-2020
(COMREC)
Research and Ethics Committee

Tidzakujambulani chifuwa chanu ndi x-ray komaso pulogalamu ya pakompyuta idzafufuza chithunziyo ngati ikusonyeza kuti pakhonza kukhala chifuwa chachikulu m'mapapo anu. Ngati pulogalamu ya pakompyuta mwawonetsa kuti mutha kukhala ndi chifuwa chachikulu tikufunsaninso kuti mukhosomoleso kuti mutulutse makhololo mu kapu yaing'ono. Kujambulidwa ndi makina a x-ray sikumapweteka nkomwe, ndipo chidzatenga nthawi yaing'ono kuti zonse zitheke.

Ngati muvomereza, tizayimbanso foni kwa inu kapena abale anu pakatha ma sabata asanu ndi zitatu (8) kuti timve mmene mukuchitira. Ngati tingalephere kukupezani palamya yanu yammanja, tizatha kukuyenderani kwanu kuti timve mmene mukuchitira.

Chimodzi mwa zoyese chifuwa chachikulu mu makhololo anu chimatenga masabta angapo kuti zotsatira zituluke. Ngati zotsatira zanu zawonetsa kuti muli ndi chifuwa chachikulu komanso simunayambe kulandira mankwala tidzakuyimbirani foni kuti mubwera kuchipatala cha chifuwa chachikulu.

Anthu onse mu kafukufukuyu adzalandira chithandizo cha HIV, chifuwa chachikulu (ngati ayenera kulandira chithandizo cha chifuwa chachikulu) ndi matenda opatsirana, mofanana ndi wina aliyense m'chipatala. Thandizo la HIV, chifuwa cha chikulu, ndi matenda opatsirana akupezeka mu Malawi komanso ndi aulere.

Kodi pali chiopsezo china chilichonse potenga nawo mbali mukafukufukuyu?
Palibe chiopsezo chachikulu chinachilichonse. Njira yojambula mu chifuwa ya x-ray ndi yotetezeka ndipo chiwopsezo chokuti njirayi itha kuyambitsa mavuto pa umoyo wanu ndichochepa kwambiri. Kuyezetsa magazi zitha kukhala zopweteka.

Kodi pali zopindula zinazilizonse potenga nawo mbali mukafukufukuyu?
Palibe chopindula chinachilichonse choonekeratu chimene inu mulandire potenga nawo mbali mukafukufukuyu. Sitikudziwabe ngati kugwiritsa ntchito chithunzi cha x-ray cha chifuwa limodzi ndi kompyuta oyang'anira zithunzizo, komanso kuyeza kwa mikozo kutha kupititsa patsogolo umoyo wa anthu; ndi chifukwa chake tikufuna kuchita kafukufukuyu. Mudzalandilabe chithandizo yoyenera chokhudzana ndi ka chirombo cha HIV, chifuwa chachikulu ndi matenda ena ngati mwasankha kapena simunasankhe kutenga nawo mbali mu kafukufukuyu.

Zokhudza chipuputa misonzi
Tizakupatsani ndalama zokwana MWK8000 potenganawo mbali mukafufukuyu.

Zokhudza chinsinsi ndi chitetezo cha mauthenga anu
Tidzateteza mauthenga anu poonetsetsa kuti tikukufunsani mafunso akafukufuku pamalo okuti palibe wina amene angamvetsere zokambirana zathu.

Anthu omwe akugwira ntchito mukafukufuku adzasunga chizindikiro ndi zotsatira zanu mwa chinsisi, kuti ndi anthu akafukufuku ndi akuchipatala amene angathe kuona mauthengawo. Tidzasunga mauthenga onse akafukufuku (kuphatikizapo chithunzi cha mchifuwa mwanu kuzera makina a x-ray) pa kompyuta yotetezedwa ndi mawu achinsinsi. Anthu ogwira nchito kuchipatala cha Zomba azakhala ndi mwayi owona chithunzi cha mchifuwa mwanu cha X-ray kuti azathe kukupasani chithandizo chamankhwala moyenerera. Ogwira ntchito kumakampani a makina a x-ray (ku Thirona ndi MinXray) azathanso kuwona zithunzizo kuti azitha kutithandiza powonesesa kuti makina a x-ray akugwila ntchito bwinolomwe. Ogwira ntchito kukampani ya makina a X-ray ya Thirona azathanso kuwona zosatila zanu za chifuwa cha chikulu cha TB kuti ziwathandize kuwonjezera mphamvu ya kopyuta kuwunika matenda a TB mwa anthu. Akadaulo a ku ma sukulu a ukachendede a London School of Hygiene and Tropical Medicine ndi College

Approved by
College of Medicine
10-Dec-2020
(COMREC)
Research and Ethics Committee

of Medicine yaku Malawi azathanso kuwona uthenga wokhuzana ndi inu, kuti ziwathandize kuwonesesa kuti kafukufukuyu akuyenda bwino.

Mauthenga amene azagawidwe adzakhala opanda chizindikiro chanu. Ndikutanthauza kuti dzina lanu silidzaphatikizidwa mu chilichonse timagawana komanso palibe amene angadziwe kuti ndi inuyo amene mwatipasa mauthengawo.

Nanga chingachitike ndi chiyani ngati mukufuna mutuluke mukafukufuku nthawi isanakwane?
Muli ndi ufulu kutuluka mukafukufukuyu pa nthawi inailiyonse yomwe inu mungafune. Ngati musakufuna kutenga nawo mbali mukafukufukuyu, kapena mwatuluka mukafukufukuyu mwamsanga nthawi yake isanakwane, ndiye kuti inu mudzathabe kulandira chithandizo china chili chonse choyenera pachipatala pano.

Tingaimbire foni ndani patakhala mafunso kapena zovuta zina?
Mutha kufunsa munthu wakafukufuku amene wakupatsani chikalatachi kapena mutha kuimbira foni a Dr Rachael Burke kapena Dr Marriott Nliwasa aku MLW pa nambala iyi +265 1 812 423 ngati muli ndi mafunso, zodandaula, kapena mwadwala kapena mwapweteka chifukwa chotenga nawo mbali mukafukufukuyu. Muthanso kuimbira mkulu wa bungwe COMREC Secretariat, College of Medicine, P/Bag 360, Chichiri, Blantyre 3, Malawi; Nambala ya lamya: +265 1871911 ext. 334; Kotumizira ma uthenga a imelo: comrec@medcol.mw

Kodi nditha kufunsa mafunso?
Muli omasuka kufunsa mafunso amene muli nawo nthawi inailiyonse. Ngati munthu wakafukufuku amene anakupatsani uthengawu sangathe kukuyankhani mafunso anu, ndiye kuti muli ololedwa kuwaimbira foni a Dr Rachael Burke kapena Dr Marriott Nliwasa pa +265 1 812 423

Chingachitidwe chiyani ngati chinachake chalakwika?
Sukulu yaukadaulo (University), The London School of Hygiene and Tropical Medicine, amakhala ndi inshuwalansi yomwe amapezeka pa kafukufukuyu. Ngati mukapwetekedwa kapena mukavulazidwa chifukwa cha kutenga nawo mbali mu kafukufukuyu, mukhoza kulandira chipepesa popanda kuwonetsa kuti sukulu yaukadaulo ndiwolakwitsa. Izi sizikhudza ufulu wanu walamulo kuti mulandira chipepets.

Approved by
College of Medicine
10-Dec-2020
(COMREC)
Research and Ethics Committee

Participant ID:

**Computer Assisted Screening for Tuberculosis in Low-resource Environments (the CASTLE study)**
**Mkulu wakafukufuku :** Dr Rachael Burke, Malawi-Liverpool-Wellcome Trust Clinical Research Programme. PO Box 30096, Chichiri, Blantyre 3, Malawi. Tel +265 1 812 423 www.mlw.medcol.mw
**Mkulu wa bungwe loyang'anira kafukufuku la COMREC** : Dr YB Mlombe, The College of Medicine of Malawi, PO Box 360, Chichiri, Blantyre 3, Malawi. Tel +265 1 871 911. www.medcol.mw

KALATA YA CHILOLEZO CHOTENGA NAWO MBALI MUKAFUKUFUKU: VERSION 4.0 2020-07-30, diagnostic cohort group

Chongani mabokosi onse

1. Ndalandira ndipo ndawelenga zokhudza kafukufuku/ ndipo opanga kafukufukuyu afotokoza momveka bwino zolinga zakafukufukuyu (CASTLE PIL Cohort v4.0 2020-07-30)	
2. Ndamvetsetsa kuti zochitika izi ndi zakafukufuku.	
3. Ndafunsa mafunso onse amene ndinali nawo okhudza cholinga chakafukufukuyu ndipo ndili okhutira kuti ndili ndi uthenga onse ofunika okhudza kafukufukuyu.	
4. Ndikuvomereza kuti, ngati ndavomera kutenga nawo mbali mu kafukufukuyu, akafukufuku ndi akadaulo ena ochokera kusukulu yaokachenjede ya College of Medicine ndi London School of Hygiene and Tropical Medicine adzayang'ana ma rekodi anga azachipatala.	
5. Ndikuvomereza kuti, ngati ndavomera kutenga nawo mbali mu kafukufukuyu, ma uthenga okhudzana ndi ine atha kuzagawidwa kwa akumakampani a x-ray (a Thirona ndi MinXray)	
6. Ndikuvomereza kuti, ngati ndavomera kutenga nawo mbali mukafukufukuyu, akafukufuku atha kusunga masampo anaga amagazi kuti azathenso kupanga makafukufuku ena a TB mtsogolomu.	
7. Ndikuvomereza kuti, ngati ndavomera kutenga nawo mbali mu kafukufukuyu, akafukufuku atha kutumiza masampo anga kunja kutiakayezedwe makani omwe kuni kulibe	
8. Ndikuvomereza kuti, ngati ndavomera kutenga nawo mbali mu kafukufukuyu, akafukufuku atha kundiimbira inuyo kapena achibale anu foni kuti tilankhulane kapena kundiyendera kwathu.	
9. Ndikudziwa kuti mauthenga onse okhudzana ndi ine adzasungidwa mwachinsinsi ndi mosadziwika kuti uthengawu wachokera kwa ine.	
10. Ngati sindingavomereze kutenga nawo mbali mu kafukufukuyu, ndamvetsetsa kuti sindidzaweruzidwa ndi akafukufuku kapena akuchipatala m'tsogolomu.	
11. Ndamvetsetsa kuti ndithakusiya kutenga nawo mbali mukafukufukuyu nthawi inailiyonse popanda chilango.	

Ngati muli ndi funso linalilionse nthawi ina, chonde yankhulani ndi mkulu wa kafukufukuyu (Dr Rachael Burke), kapena mkulu wa bungwe COMREC pa adiresi ndi nambala za foni zilipamwambazo

Ndalolera mwakufuna kwanga kutenga nawo mbali mukafukufukuyu

**Dzina lanu, (MUZILEMBO ZAZIKULU)**	**Tsiku (DD-MON-YYYY)**	**Sayini kapena chidindo cha chala**
**Dzina la mboni (MUZILEMBO ZAZIKULU) (Ngati satha kulemba/ kuwerenga)**	**Tsiku (DD-MON-YYYY)**	**Sayini**
**Dzina la wakafukufuku(MUZILEMBO ZAZIKULU)**	**Tsiku (DD-MON-YYYY)**	**Sayini**

Sayinani pa mapepala awili: pepala limodzi mupatse otenga nawo mbali mukafukufuku. Ina yokuti isungidwe limodzi ndimapep

Approved by
College of Medicine
10-Dec-2020
(COMREC)
Research and Ethics Committee
