# Supplementary material for: Design and protocol for a cluster randomised trial of enhanced diagnostics for tuberculosis screening among people living with HIV in hospital in Malawi (CASTLE study)
Source: PLoS One. 2022 Jan 10;17(1):e0261877. doi: 10.1371/journal.pone.0261877 (PMC8746787; doi:10.1371/journal.pone.0261877)
Supplement: S2 Appendix — (PDF) [file pone.0261877.s003.pdf]

The CASTLE study

Computer Aided Screening for Tuberculosis in Low Resource Environments

Document: Data Monitoring and Safety Committee Charter

Version: 1.0

Date last updated: 2019-08-27

1 Introduction

1.1 Trial name

Computer Aided Screening for Tuberculosis in Resource Limited Environments (CASTLE)

1.2 Trial registration

The study will be registered with ISRCTN before commencement.

1.3 Ethics reference number

The CASLTE study has received ethical review as follows;

LSHTM Research Ethics Committee. Reference 17799.

University of Malawi College of Medicine Research and Ethics Committee (COMREC). Reference: P.08/19/2772.

1.4 Sponsor and Funder

The London School of Hygiene and Tropical Medicine is the main research sponsor for this study. For further information regarding the sponsorship conditions, please contact the Research Governance and Integrity Office:

London School of Hygiene and Tropical Medicine
Keppel Street
Lonwon WC1E 7HT
+44 2079272626
rgio@lshtm.ac.uk

The funder is the Wellcome Trust, via a Wellcome Trust Clinical PhD Fellowship awarded to Dr Rachael Burke.

1.5 Investigators

A full list of investigators is listed in the protocol. The principal investigator is Dr Rachael Burke (a PhD candidate). Her PhD supervisors and co-investigators are Prof Liz Corbett and Dr Peter MacPherson. Prof Katherine Fielding is a co-investigator.

1.6 Scope of charter

The purpose of this document is to describe the roles and responsibilities of the Trial Monitoring Committee for the CASTLE trial, including the timing of meetings, methods of providing information, frequency and format of meetings, statistical issues and relationships with other committees.

2 Trial summary (please also refer to trial protocol)

2.1.1 Background

Despite the widespread availability of ART at community level, people living with HIV (PLHIV) who require admission to hospital in WHO AFRO region are frequently very immunosuppressed and have

a high mortality. Undiagnosed TB is likely to be a major contributor to mortality. Suboptimal TB diagnostics contribute to the problem.

Digital chest X-ray with computer aided diagnosis (DCXR-CAD) is a promising new technology to assist with X-ray interpretation, particularly in a setting where skilled radiologists are not routinely available. Fujifilm SILVAMP TB LAM (FujiLAM) is a CE-marked high sensitivity urine LAM test. Retrospective studies on stored samples have shown superior sensitivity of FujiLAM compared to the older urine LAM test manufactured by Alere / Abbott.

Our trial hypothesis is that admission screen for TB among adults living with HIV using DCXR-CAD and FujiLAM plus usual care can increase the number of people starting on TB treatment and reduce inpatient mortality, compared to usual care alone.

A more detailed summary of scientific background with references is available in the protocol.

2.1.2 Summary

Trial title	Computer Aided Screening for Tuberculosis in Low Resource Environments (CASTLE)
Short title	CASTLE study
Trial Design (methodology)	Single site (Zomba Central Hospital) cluster randomised trial with two trial arms and a third nested observational enhanced diagnostic cohort that will not contribute to trial outcomes (4:4:1 allocation, randomised by admission day).
Trial population	HIV infected adult patients requiring admission to medical wards at Zomba Central Hospital. Unit of randomisation will be admission day.
Planned sample size	102 clusters per trial arm (approximately 306 participants). A further 26 clusters in enhanced diagnostic cohort (approximately 78 participants). Total of 230 clusters with approximately 690 participants..
Follow up duration	56 days (eight weeks) from day of recruitment
Recruitment period	January 2020 – March 2021.
Trial intervention	Digital Chest x-ray with Computer Aided Diagnosis (DCXR-CAD) and urine high sensitivity lipoarabinomannan (FujiLAM) screening performed on first day of admission on participants admitted on days assigned to trial intervention arm. Numerical X-ray TB score and interpretation ("Pulmonary TB likely" or "Pulmonary TB not likely"), and FujiLAM results, appended into patient's notes. X-ray imaging available for clinical team review on study computer in order to inform TB treatment decision making. If a participant's CAD score indicates "TB likely", they will have sputum taken for Xpert Mtb/Rif. DCXR-CAD is in addition to usual care in the intervention arm. The control arm is assigned to usual care alone.

2.1.3 Objectives and outcomes

	Objective	Outcome Measures / Endpoints
1. Primary	To determine the effect of DCXR-CAD plus FujiLAM plus usual care vs. usual care alone on;<br>1.1 TB treatment initiations	1.1 Proportion of participants starting TB treatment during course of inpatient stay (censored at 56 days)
2. Secondary	To determine the effect of DCXR-CAD plus FujiLAM plus usual care vs. usual care alone on;<br><br>2.1 Mortality (time to event)<br>2.2 Undiagnosed TB	2.1 Time (in days) to death from any cause, with censoring at 56 days.<br><br>2.2 Proportion of participants who are culture positive for *M. tuberculosis* (M.tb) in sputum, who are not started on TB treatment at the time of discharge

	2.3 Same day TB treatment initation	from hospital or are current inpatients not on TB treatment by the time of culture result being made available.<br>2.3 Proportion of participants starting TB treatment within 24 from from time of recruitment.
3.Pre-planned analyses.	To determine the effect of DCXR-CAD plus FujiLAM plus usual care vs. usual care alone on;<br>3.1 Inpatient mortality (proportion of participants dying as inpatients).<br><br>3.2 Total mortality (proportion experiencing death)<br><br>3.3 Proportion of participants with a TB diagnosis that is microbiologically confirmed vs. clinically diagnosed.<br><br>3.4 To determine intervention fidelity in this setting.<br><br>3.4 To determine diagnostic accuracy of DCXR-CAD in an inpatient, HIV positive setting.	3.1 Proportion of participants dying during admission (censored at 56 days) from any cause.<br><br>3.2 Proportion of participants dying up to 56 days from any cause.<br><br>3.3 Proportion of participants started on TB treatment with (a) microbiologically confirmed TB vs. (b) clinically or radiologically diagnosed TB.<br><br>3.4 Proportion of participants randomised to DCXR-CAD plus FujiLAM arm who received DCXR-CAD and had a urine FujiLAM result.<br><br>3.4 Sensitivity, specificity, PPV and NPV for CAD score compared to a composite microbiological reference standard and a clinical standard.
4. Diagnostic cohort	To describe the range of pathology among people living with HIV requiring admission to hospital	Descriptive statistics.

2.1.3.1 *Trial schematic*

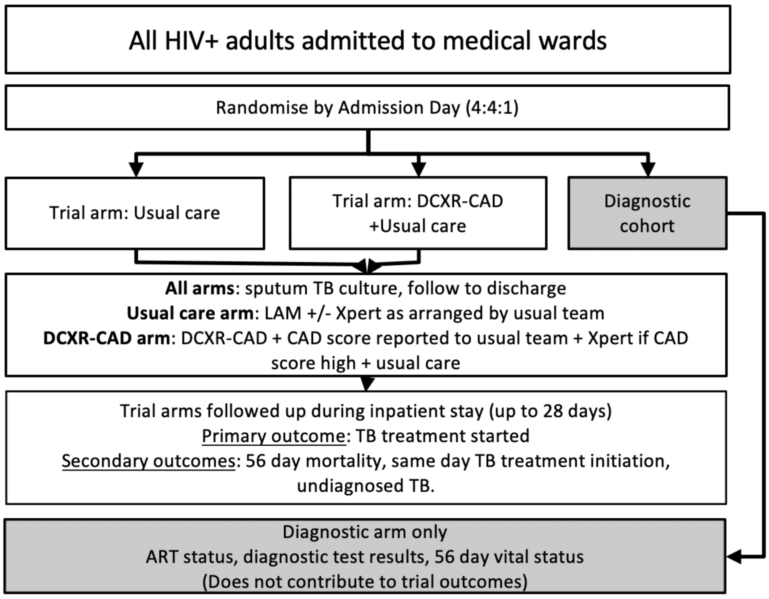

3 Roles and responsibilities

3.1 Aims of the DSMC committee

The aim of the DSMC is to protect the safety of study participants, to assist and advise the Principal Investigator (Dr Rachael Burke, a PhD student), co-investigators (including Prof Liz Corbett and Dr Peter MacPherson, her PhD supervisors) and collaborators so as to protect the validity and credibility of the trial, and to monitor the overall conduct of the clinical trial. The DSMC will also provide advice through its chairperson to the sponsor of the trial.

The DSMC will be a single body which will take on the joint roles of a traditional Trial Steering Committee and a Data Safety and Monitoring Board.

A full list of co-investigators and collaborators is listed on the CASTLE study protocol.

3.2 Terms of reference

The DSMC will

1) Receive and review the progress and accruing trial data (by default, blinded data only but the independent members may request to review unblinded data in a closed session);
2) Advise the principal investigator and co-investigators on the conduct of the trial;
3) Review and comment on the statistical analysis plan.

3.3 Specific roles of the DSMC

1) Provide expert oversight of the trial
2) Monitor recruitment rates and encourage investigators to develop strategies to deal with any recruitment problems
3) Assess the impact and relevance of any accumulating external evidence
4) Advise on protocol modifications relevant to data aspects as suggested by investigators or sponsors (eg. to inclusion criteria, trial endpoints, or sample size)
5) Review amendments to the protocol, where appropriate
6) Review the statistical analysis plan
7) Encourage the timely reporting of trial results
8) Monitor adverse events (see protocol for specific adverse events which will be reported)
9) Maintain confidentiality of all trial information that is not already in the public domain

4 Before the trial starts

The DSMC membership will review and provide comments on the trial protocol, and hold their first meeting before commencement of randomisation of participants. The objective of the first meeting is to discuss the protocol including adverse event reporting and analysis plans with the principal investigator and co-investigators.

The first meeting will also involve planning future meetings and reviewing what the contents of the data report should be (dummy tables in annex 3 and 4).

5 Composition

The members of the DSMC for the CASTLE trial are:

1)

2)

3)

4)

The DSMC membership includes statistical and clinical expertise. ________ will be the chairperson.

As this trial is her PhD project, the Principal Investigator will act as the facilitator of the DSMC. She will be responsible for arranging meetings of the DSMC, coordinating reports, producing and circulating minutes and action points. The PI / facilitator will be the central point for all DSMC communications between the DSMC and other bodies, will be copied into all correspondence between DSMC members and will be kept aware of trial issues as they arise.

DSMC members will not be asked to formally sign a contract but should formally register their agreement to join the group by confirming (1) that they agree to be a member of the DSMC and (2) that they agree with the contents of this Charter. Any potential competing interests should be declared at the same time. Members should complete and return the form in Annexes 1 or 2.

Additional observers may be in attendance through (parts of) the DSMC meetings in order to provide input on behalf of the trial's Sponsor/Funder or to provide specific relevant expertise.

6 Preparation of reports to the DSMC

The PI will prepare the open report.

It is anticipated that all committee meetings will be open. The first meeting will be before the commencement of the trial, thus a closed portion to this meeting is unnecessary.

By default, the interim meeting(s) during the course of the trial will also be open. However, at the committee's request, a closed session will be convened consisting only of the independent members of the committee and a statistician representative from the trial (Prof Fielding). At this closed session unblinded data could be presented and discussed.

If the independent members of the DSMC request to view the unblinded data in a closed session then the PI will write the statistical analysis code which will be used for the data analysis for the closed report. She will not run the data analysis for the closed report so as to remain blinded. The statistical code will be run and the report will be compiled by an independent statistician from Malawi Liverpool Wellcome Clinical Research Programme.

7 Relationships

Trial management is described in the protocol.

The DSMC has an advisory role for the trial.

DSMC members will not be paid for their services.

DSMC members are asked to disclose information about any competing interests.

Formally, the DSMC reports to the sponsor (the London School of Hygiene and Tropical Medicine) and their meeting reports will be available to the sponsor. Excepting major issues, we envisage the comments from the DSMC will be taken forward for action by the principal investigator and/or her supervisors / co-investigators.

8 Organisation of DSMC meetings

DSMC meetings will be conducted once prior to commencement of trial, once during the trial (after 6 – 9 months) and once at the conclusion of the trial (12-15 months). Should issues arise during the course of the trial, the DSMC will meet more frequently – either at the request of the PI or co-investigators or at the request of the independent members of the committee.

The meetings will be conducted by teleconference.

By default, the meeting will have only an open session. However, should the DSMC request a closed session to review unblinded data then a meeting with a closed session will be convened.

Attendance by the study investigational team (with the exception of Prof Fielding) will be restricted to the open session – i.e. if there were to be a closed session this would be for will be for independent DSMC members and Prof Fielding only.

9 Trial documentation and procedures to ensure confidentiality and proper communication

An outline of the intended content of material to be available in open sessions will be prepared and agreed at the first DSMC meeting.

The DSMC will receive the reports at least 1 week before any meetings.

The PI will take minutes of the open session. Should a closed session be convened Prof Fielding could make brief notes, alternatively - if requested - the PI would arrange for an administrator to take minutes.

The DSMC will report its recommendations in a written report, which will be copied to the sponsor. It is anticipated that the actions from meeting will be taken forward by the principal investigator.

Where the independent members of the DSMC have met in a closed session, they will present their recommendations to the rest of the DSMC (ie. the principal investigator and co-investigators) and these will be included in the written report.

10 Decision making

The DSMC may make recommendations such as:

1) No action needed, trial continues as planned
2) Modifying target recruitment, or pre-analysis follow-up, based on any change to the assumptions underlying the original trial sample size calculation (but not on any emerging differences)
3) Sanctioning and/or proposing protocol changes

4) Early stopping due, for example, to clear benefit or harm of a treatment, futility or external evidence

Every effort should be made to achieve consensus. The role of the Chair is to summarise discussions and encourage consensus.

It is important that the implications (e.g. ethical, statistical, practical, financial) for the trial be considered before any decision is made.

10.1 When is the DSMC quorate for decision-making?

At least two independent members of the DSMC should be present, plus the PI.

The PI (who is also the DSMC facilitator) will, ahead of a planned meeting, coordinate with all DSMC members and identify an agreeable meeting date. Two independent members unless otherwise agreed) can still run a DSMC meeting. If the DSMC is considering recommending major action after such a meeting the chair should talk with the absent members as soon after the meeting as possible to check if they agree. If they do not, a further teleconference should be arranged with the full DSMC. If the report is circulated before the meeting, DSMC members who will not be able to attend the meeting may pass comments to the DSMC Chair for consideration during the discussions.

If a member does not attend a meeting, it will be ensured that the member is available for the next meeting. If a member does not attend a second meeting, they will be asked if they wish to remain part of the DSMC. If a member does not attend a third meeting, they will cease to be a DSMC member.

11 Reporting

11.1 To whom will the DSMC report their recommendations/decisions, and in what form?

The DSMC will report their decisions to the PI and co-investigators who will be responsible for implementing any actions resulting.

The DSMC report will be copied to the sponsor as a matter of course. Should major action be recommended, this will be highlighted to the sponsor.

11.2 Whether minutes of the meeting be made and, if so, by whom and where they will be kept

Notes of key points and actions will be made by the PI (who also acts as the DSMC facilitator). This will include details of whether potential competing interests have changed for any attendees since the previous meeting. The draft minutes will be initially circulated for comment to those DSMC members who were present at the meeting. The DSMC Chair will sign off the final version of minutes or notes.

11.3 What will be done if there is disagreement within the DSMC (particularly between the investigators and the independent members) ?

The DSMC is the oversight body for the trial and combines the traditional roles of TSC and DSMB into one committee.

Should there be disagreement within the DSMC, the committee would first try to resolve the issue internally by consensus, under the guidance of the independent chairperson.

In exceptional circumstances, if the matter could not be resolved by consensus, the DSMC chair may contact the trial sponsor who may convene a meeting chaired by a senior member of LSHTM or an external expert who is not directly involved with the trial. Depending on the reason for the disagreement confidential data and/or data by trial and may have to be revealed to all or some of those attending such a meeting: this would be minimised where possible.

12 After the trial

DSMC members will be named and their affiliations listed in trial report, unless they explicitly request otherwise. A brief summary of the timings and conclusions of DSMC meetings may also be included in this report.

13 Annex 1: Trial Monitoring Committee members register of their assent

I, __________________ agree

1) to be on the CASTLE Trial DSMC committee
2) with the contents of the CASTLE Trial DSMC Charter
3) to keep CASTLE Trial DSMC data reports and meeting outputs confidential

Signature: __________________________________ Date: _______________

14 Annex 2: Suggested competing interests form

Potential competing interests of DSMC

Possible competing interest(s) should be disclosed.

Potential competing interests

- Stock ownership in any commercial companies involved
- Stock transaction in any commercial company involved (if previously holding stock)
- Consulting arrangements with the sponsor
- Frequent speaking engagements on behalf of the intervention
- Career tied up in a product or technique assessed by trial
- Hands-on participation in the trial
- Involvement in the running of the trial (for independent members only, this conflict is not relevant to members who are on the DSMC by virtue of their involvement with running the trial)
- Intellectual conflict eg. strong prior belief in the trial's intervention arm
- Involvement in regulatory issues relevant to the trial procedures
- Investment (financial or intellectual) in competing products
- Involvement in the publication

Please complete the following section and return to the Principle Investigator.

☐ **No,** I have no competing interests to declare

☐ **Yes,** I have competing interests to declare (please detail below)

Please provide details of any competing interests:

Name: ___________________________

Signed: ___________________________ Date: _______________

15 Annex 3: Data for open session

Data will not indicate study arm and the following will be presented

- Enrolment and accrual

Study week (4 clusters per week)	Number potential participants screened	Number enrolled (total in 4 clusters for that week)	Running total clusters and participants

- Participant retention

Number of participants reached 56 day follow up	Number reached to ascertain outcome	Comments on strategies to reach participants

- Any participants withdrawn?

- Participant baseline characteristics

Characteristics	Median (IQR) or proportion
Age	
Sex	
On ART or not	
TB suspected at admission?	

- In hospital deaths observed

n in hospital deaths	n people discharged from hospital alive (or alive in hospital and >56 days from enrollment)	n admitted to hospital

- Deaths by 56 days

n community deaths by 56 days from enrolment	n people known to be alive at 56 days	n participants not yet reached 56 days from enrolment	n participants >56 days from

			enrolment but not reached

- TB treatment initiations

n TB treatment initiations	N participants

- TB sputum *culture* results (NB. Does not include TB tests (eg. Xpert) done locally)

	N participants
N participants	
Number (%) sputum samples produced and received in lab	
Number (%) culture positive	
Number (%) culture negative	
Number (%) culture ongoing	
Number (%) contaminated / otherwise unavailable results.	

- Adverse events

	Number of events
NB. Inpatient death reported as a trial outcome (see above)	
Error TB results reporting leading to participant starting TB treatment in error	
Breach of confidentiality following TB or HIV diagnosis	
Needlestick injuries	

Further details about circumstances of adverse events (if required)

- Have the investigators become aware of any participants who were diagnosed with TB, but were later discovered to have a diagnosis other than TB? If details of cases to be outlined below.

Participants who were diagnosed with TB initially, but later discovered to have a non-TB diagnosis

16 Annex 4: Data for closed session

Part 1: Aggregate data presented with statistical comparisons (for determining safety)

- TB treatment initiation by study arm

Arm 1 (usual care)	Arm 2 (DCXR-CAD and FujiLAM intervention)	Arm 3 (diagnostic cohort)	Arm 1vs arm 2 95% CI, p-value	Arm 1 vs arm 3 95% CI, p-value

- Mortality by study arm

Arm 1 (usual care)	Arm 2 (DCXR-CAD and FujiLAM intervention)	Arm 3 (diagnostic cohort)	Arm 1vs arm 2 95% CI, p-value	Arm 1 vs arm 3 95% CI, p-value

- LTFU (withdrawl) by study arm

Arm 1 (usual care)	Arm 2 (DCXR-CAD and FujiLAM intervention)	Arm 3 (diagnostic cohort)	Arm 1vs arm 2 95% CI, p-value	Arm 1 vs arm 3 95% CI, p-value

Part 2: Within intervention arm comparisons of diagnostic performance

	**CAD score below threshold**	**CAD score above threshold**
All		
Sputum produced for Xpert (study team)		
Xpert positive (study Xpert)		
TB treatment initiation (n/N)		
% TB treatment initiations that are empiric (without microbiological confirmation)		

	**AlereLAM positive**	**AlereLAM negative**	
**FujiLAM positive**			
**FujiLAM negative**			
			N =<br>Total with urine sample and valid LAM results

Those who are FujiLAM positive and AlereLAM negative (N=)		
**Study sputum culture**		
	Not done	
	Positive	
	Negative	
**CAD score**		
	Not done	
	Above threshold	
	Below threshold	
**Study Xpert**		
	Not done (NB. Not mandated if CAD score below threshold)	
	Positive	
	Negative	
**TB treatment initiation**		
	Yes	
	No	
